# Delta-S-Cys-Albumin: A Lab Test that Quantifies Cumulative Exposure of Archived Human Blood Plasma and Serum Samples to Thawed Conditions*[Fn FN2]

**DOI:** 10.1074/mcp.TIR119.001659

**Published:** 2019-07-19

**Authors:** Joshua W. Jeffs, Nilojan Jehanathan, Stephanie M. F. Thibert, Shadi Ferdosi, Linda Pham, Zachary T. Wilson, Christian Breburda, Chad R. Borges

**Affiliations:** ‡ School of Molecular Sciences and The Biodesign Institute at Arizona State University, Tempe, AZ 85287; § Maricopa Integrated Health System, Phoenix, AZ 85008; ¶ University of Arizona College of Medicine, Phoenix, AZ 85004

**Keywords:** Serum/plasma, plasma or serum analysis, biobanking, quality control and metrics, post-translational modifications, cysteine, cystine, quality assurance, s-cysteinylated albumin

## Abstract

Exposure of blood plasma/serum (P/S) to thawed conditions (> −30 °C) can produce biomolecular changes that skew measurements of biomarkers within archived patient samples, potentially rendering them unfit for molecular analysis. Because freeze-thaw histories are often poorly documented, objective methods for assessing molecular fitness before analysis are needed. We report a 10-μl, dilute-and-shoot, intact-protein mass spectrometric assay of albumin proteoforms called “ΔS-Cys-Albumin” that quantifies cumulative exposure of archived P/S samples to thawed conditions. The relative abundance of S-cysteinylated (oxidized) albumin in P/S increases inexorably but to a maximum value under 100% when samples are exposed to temperatures > −30 °C. The difference in the relative abundance of S-cysteinylated albumin (S-Cys-Alb) before and after an intentional incubation period that drives this proteoform to its maximum level is denoted as ΔS-Cys-Albumin. ΔS-Cys-Albumin in fully expired samples is zero. The range (mean ± 95% CI) observed for ΔS-Cys-Albumin in fresh cardiac patient P/S (*n* = 97) was, for plasma 12–29% (20.9 ± 0.75%) and for serum 10–24% (15.5 ± 0.64%). The multireaction rate law that governs S-Cys-Alb formation in P/S was determined and shown to predict the rate of formation of S-Cys-Alb in plasma and serum samples—a step that enables back-calculation of the time at which unknown P/S specimens have been exposed to room temperature. A blind challenge demonstrated that ΔS-Cys-Albumin can detect exposure of groups (*n* = 6 each) of P/S samples to 23 °C for 2 h, 4 °C for 16 h, or −20 °C for 24 h—and exposure of individual specimens for modestly increased times. An unplanned case study of nominally pristine serum samples collected under NIH-sponsorship demonstrated that empirical evidence is required to ensure accurate knowledge of archived P/S biospecimen storage history.

## Abstract (precis)

ΔS-Cys-Albumin, an endogenous marker of plasma and serum (P/S) exposure to thawed conditions (> −30 °C) based on the *ex vivo* S-cysteinylation (oxidizability) of albumin was developed. Average values in fresh P/S samples from a population of nonacute cardiac patients were determined. The multireaction mechanism that drives changes in albumin S-cysteinylation is known and the rate law for it has been established and accurately modeled in P/S. Measurement of ΔS-Cys-Albumin in unknown samples facilitates estimation of thawed-state exposure times.

## Graphical Abstract

**Figure:**
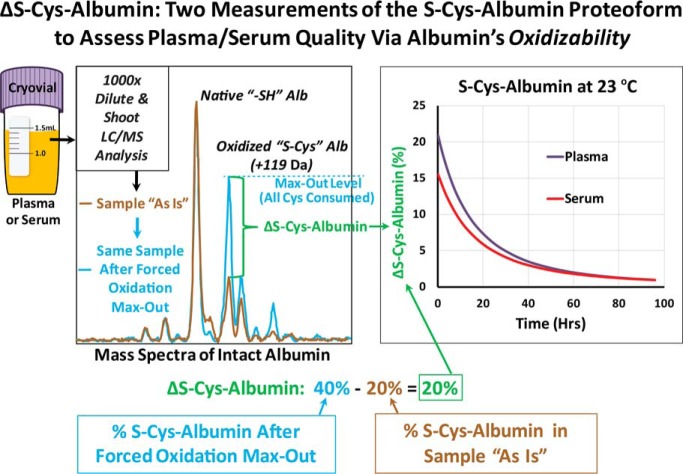

**Highlights**

- Endogenous plasma/serum QC marker that quantifies cumulative sample thawed time.
- Mechanism of marker change known, and rate law established.
- Data interpretation based on documented population averages and rate law.
- Blind-challenge verified; utility proven via exposure of undisclosed freezer outage.

Collection, processing, storage and handling expose clinical biospecimens to pre-analytical variables that, when unaccounted for, have the potential to produce misleading results in downstream clinical research (1–5). For blood plasma and serum (P/S)^1^ samples, numerous pre-analytical variables such as the type of collection tube, degree of tube filling, number of tube inversions, degree of hemolysis, and pre-centrifugation delay can quantitatively impact clinical measurements (6–9). Arguably, however, exposure to the thawed state (*i.e.* temperatures > −30 °C (10, 11)) is the most difficult pre-analytical variable to track and control over the lifetime of a P/S biospecimen—particularly at the individual aliquot level.

There is widespread agreement regarding the need for robust biospecimen quality control (QC)/quality assurance (QA) checks in biomarker discovery and validation work. Yet relatively little research effort focuses on this arena. P/S specimens are among the most commonly employed biospecimens in biomarker-related research but, to date, no gold standard marker of P/S integrity has been identified and put into widespread, routine use. This is evidenced by the fact that despite the recent emphasis on robustness and reproducibility, the U.S. National Cancer Institute (NCI, part of the National Institutes of Health) does not currently require any empirical evidence-based QA thresholds be met before pre-existing P/S specimens are employed in NCI-sponsored research. This QC/QA problem is widespread: In 2014, for example, out of 455 NCI-sponsored extramural grants that involved biospecimens, 287 (63%) relied on pre-existing samples; over 100 of these sponsored projects employed pre-existing P/S (12).

Written documentation that includes the types of specimens analyzed and the specimen storage conditions is now required for manuscript submissions to leading clinical research journals (13–15), but as we have experienced—and describe herein—paper trails are insufficient to guarantee disclosure of incidents that may compromise specimen integrity. The possible reasons for this are rarely discussed but may range from poor note taking to conflicts of interest with disclosing incidents that may have resulted in biospecimen damage. Moreover, paper trails lack the ability to rigorously quantify the molecular integrity of specimens that may have experienced minor “exposure” incidents that are either not captured in the documentation or, if they were, cannot be precisely assigned to individual samples or aliquots. Yet if temperature-unstable or even *potentially* temperature-unstable markers are to be analyzed in a set of P/S samples, it is critically important to know the biomolecular integrity of every sample.

This line of reasoning implies that a gold standard metric of P/S integrity should involve some form of empirical, quantitative biomolecular analysis that, if necessary, could be applied to every sample in a clinical study (whether the sample was collected prospectively or is to be analyzed retrospectively)—even if the study employed thousands of P/S samples. As such, the ideal assay would focus on endogenous analyte(s), require a very low sample volume, require minimal sample preparation, be automatable from the point of fully frozen P/S to the point of generating the final report, and be both inexpensive and rapid. Moreover, the majority, if not all, of the representative molecular alterations caused by unavoidable (bio)chemical processes that occur on P/S exposure to the thawed state should be captured by the integrity marker. Such processes include (1) drifting toward an equilibrium state that is never actually reached *in vivo*, (2) *ex vivo* oxidation (because of P/S samples taking on a dissolved oxygen concentration of up to 0.25 mm (16, 17)—a concentration that is orders of magnitude higher than that observed in the P/S compartment *in vivo* where oxygen is primarily carried on hemoglobin inside red blood cells), (3) enzyme-mediated biomolecular degradation, and (4) macromolecular denaturation. Additionally, changes in the QA marker(s) should occur in the same time frame in which some of the most “fragile” time-sensitive biomolecules within the P/S sample are altered beyond their original *in vivo* status. And finally, if intended as generally representative, thawed-state sensitive changes in QA marker(s) should neither be inhibited nor accelerated by stabilization practices that focus on a single mechanism of *ex vivo* alteration such as protease inhibitors or heat-based “inactivation.”

Together, these characteristics are quite stringent and are unlikely to be met by a single QA analyte or assay. As such, it should at least be possible to link changes in the QA marker to changes in specific analytes of interest by analyzing both concurrently. When linked empirically, this is possible for most imaginable QA markers. However, the best QA markers will be those for which a (bio)chemical rate law can be determined. This will make it possible for the marker(s) to serve as a molecular stopwatch for the timespan of thawed-state exposure and effectively place an exposure time stamp on each sample. With the rate law for the QA marker established, it would be possible for any investigator to link their assay(s) of interest to the QA marker by simply following good assay development guidelines and conducting a stability time course with their clinical analyte(s) of interest.

In 2014 we reported that two of the most abundant P/S proteins, albumin and apolipoprotein A-I, were susceptible to *ex vivo* oxidation events that occur over two nonoverlapping time segments in P/S specimens exposed to temperatures > −30 °C—yet the proteins were stable when P/S was kept at −80 °C. These proteins were measured in a single dilute-and-shoot LC-MS assay of the intact proteins that required less than 1 μl of P/S (18). It was clear that albumin oxidation (S-cysteinylation of its single free cysteine residue) would meet most of the ideal QA marker specifications described above, but the *in vivo* reference range for the percentage of albumin in this oxidized state and the multireaction rate law for the formation of S-cysteinylated albumin were not known. Subsequently (and as reported herein), we made measurements that provided an estimate of the population reference range for the fraction of albumin in its S-cysteinylated form (S-Cys-Alb), but found that it was close to the maximum degree of S-cysteinylation obtained by some samples *ex vivo*—*i.e.* once albumin had consumed all of the free cysteine (Cys) and cystine (Cys-Cys) equivalents present. This limited, in theory, the useable dynamic range of S-Cys-Alb as a P/S QA marker. Nevertheless, the population reference range for albumin in P/S is known (19) and those for Cys and Cys-Cys in plasma are well estimated (20, 21). Thus assuming that albumin is the only significant oxidative consumer of Cys equivalents, it is possible to calculate that if S-Cys-Alb is measured in a fresh plasma sample then intentionally driven to its maximum possible value *ex vivo* (18), 99% of the human population under age 60 should experience a *change* in S-Cys-Alb between these two measurements (ΔS-Cys-Alb) of 11–30%. (This range increases in persons over age 60 to 17–38% because albumin concentrations decrease (22) and Cys-Cys concentrations increase (20) with age.) Charge deconvoluted ESI-mass spectra of albumin that illustrate the ΔS-Cys-Alb phenomenon are provided in Fig. 1. Given that the inter-assay precision for measurement of S-Cys-Alb is 1.6% (18) and thus for ΔS-Cys-Alb is 2.2% (23), we intentionally pursued ΔS-Cys-Alb as a QA marker for exposure of P/S samples to the thawed state.

**Fig. 1.**
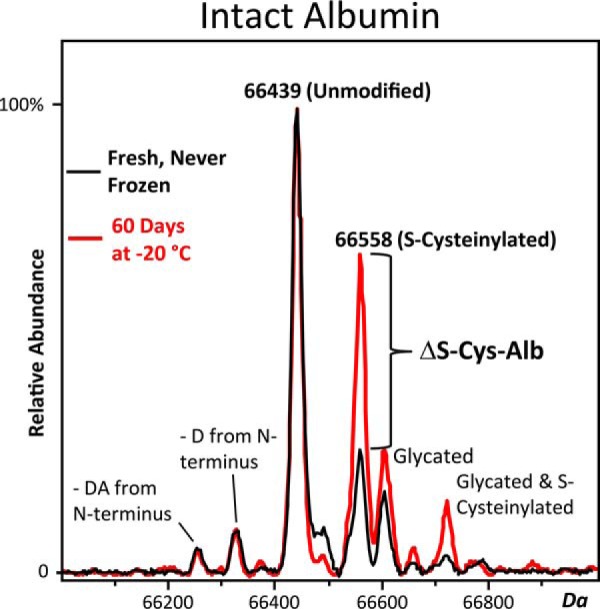
**Charge deconvoluted ESI-mass spectra of albumin that illustrate ΔS-Cys-Alb.** The black spectrum is from a fresh sample obtained immediately after centrifugation. The red spectrum is from the same sample stored for 60 days at −20 °C. The same shift occurs when P/S samples are intentionally incubated at 37 °C for 18 h. As indicated, small fractions of albumin are N-terminally truncated, and both the native and S-cysteinylated forms may be glycated. This figure was originally published in Molecular and Cellular Proteomics (18). © the American Society for Biochemistry and Molecular Biology.

Here we report development of an assay for ΔS-Cys-Alb, estimates for the population reference ranges for S-Cys-Alb and ΔS-Cys-Alb, and the multireaction rate law for formation of S-Cys-Alb at room temperature (which facilitates back-calculation of P/S sample exposure times). We also provide insights into the role of dissolved oxygen in driving S-Cys-Alb formation, the results of a blind challenge of the ability of ΔS-Cys-Alb to detect exposures of both *groups* of P/S samples and *individual* P/S samples to the thawed state (*i.e.* temperatures > −30 °C), and an unplanned case study in which ΔS-Cys-Alb detected and subsequently prompted disclosure of a biospecimen integrity discrepancy in a set of nominally pristine serum samples collected under NIH sponsorship.

## EXPERIMENTAL PROCEDURES

Detailed information on the materials and reagents employed is provided in supplemental Information.

### 

#### 

##### Plasma and Serum

Multiple collections of matched K_2_EDTA plasma and serum samples from a 41-yr old healthy male volunteer were collected, via forearm venipuncture, under informed consent and institutional review board (IRB) approval at Arizona State University. The samples were collected according to the NIH's Early Detection Research Network blood collection standard operating procedures (24, 25). All tubes were filled to the proper level and inverted the proper number of times (8 times for EDTA plasma and 5 times for serum). Within 30 min of collection, plasma samples were processed, aliquoted, and placed in a −80 °C freezer; serum samples were allowed to clot for ∼ 40 min, then immediately centrifuged, aliquoted, and placed at −80 °C within 70 min of collection.

Matched K_2_EDTA plasma and serum samples were collected under informed consent and IRB approval from nonacute cardiac patients presenting with chest pain suggestive of coronary artery disease and undergoing coronary angiogram, cardiac stress test and/or coronary computed tomography angiography at Maricopa Integrated Health System. Patients were a 40/60 mixture of females/males, ranging in age from 34–85 years (mean ± S.D. was 60 ± 9.6 years). None of the patients had severe or end-stage renal disease (*i.e.* estimated glomerular filtration rate, eGFR < 30 ml/min*1.73 m^2^) and none were on hemodialysis; only 11 had eGFR values < 60 ml/min*1.73 m^2^. Samples were collected and processed as described above for the healthy volunteer. Times of draw, centrifugation and placement at −80 °C were recorded for every individual sample. Sample hemolysis was noted by visual comparison to a color chart, resulting in placement of samples into categories of minimal, mild, moderate, and high hemolysis—corresponding to < 20 mg hemoglobin/dL, 20–50 mg/dL and 50–250 mg mg/dL and > 250 mg/dL, respectively. Samples with > 250 mg/dL hemolysis were excluded; results from such samples are not reported herein.

Serum specimens for the case study of pre-existing samples were collected under IRB approval from stage I lung cancer patients and corresponding age, gender and smoking-status matched controls. These samples were collected under NIH-sponsorship by seasoned investigators with well-defined, matched standard operating procedures. In brief, serum samples were collected into red top tubes (BD Vacutainer catalog no. 366430). These were allowed to sit upright at room temperature for 30–60 min after blood was drawn to allow the clot to form. If the blood was not centrifuged immediately after the clotting time, the tubes were refrigerated at 4 °C for no longer than 24 h. After clotting, samples were centrifuged at 1200 RCF for 20 min at room temperature. Aliquots were then placed into 2-ml cryovials and stored at −80 °C. Most case and control samples were processed and placed at −80 °C within 2–3 h of collection. Cancer patients were 69% female/31% male and controls were 71% female/29% male. The average age (mean ± S.D.) of the cancer patients and controls was 71.9 ± 9.2 years and 68.4 ± 8.1 years, respectively. Cancer patients had a slightly lower average number of smoking pack years (33.9 pack years) compared with the controls (37.3 pack years).

All P/S samples were coded and de-identified before transfer to the analytical laboratory. All human subjects experiments were conducted according to the principles expressed in the Declaration of Helsinki.

##### S-Cys-Alb and ΔS-Cys-Alb Assay

Sample Preparation: P/S samples are prepared for injection onto the LC-MS by 1000x dilution in 0.1% (v/v) TFA. Typically, 0.5 or 1 μl was mixed with 500 or 1000 μl of 0.1% TFA. (Once diluted, S-Cys-Alb measurements are stable for over 16 h at 10 °C (18).) For measurement of ΔS-Cys-Alb, 9 μl of residual P/S sample was then placed into a 0.6-ml Eppendorf snap-cap polypropylene test tube and incubated in an oven set at 37 °C for 18 h. Following this period, the sample was diluted 1000× in 0.1% TFA then injected onto the LC-MS. The *difference* in S-Cys-Alb before and after this 18-h incubation at 37 °C constitutes ΔS-Cys-Alb. Notably, for all measurements of S-Cys-Alb, matched plasma and serum samples were prepared by the same analyst and analyzed one right after another (interspersed) on the LC/MS instrument.

##### LC-ESI-MS

We have previously reported the LC-MS procedure to measure the fraction of albumin that is S-cysteinylated (S-Cys-Alb), including autosampler stability (18). Detailed LC-MS and data analysis settings are provided in supplemental Information.

##### Rate Law Determination

The rate law for formation of S-Cys-Alb in the P/S environment is governed by the chemical reactions listed in Eqs. 1–2 (see Results section). The rate law for Eq. 2 was previously determined by Kachur *et al.* (26). Thus, to obtain a complete combined rate law for formation of S-Cys-Alb, the rate laws for the forward and reverse reactions described in Eq. 1 were determined using the method of initial rates (27). Detailed methodological information is provided in supplemental Information.

##### Statistical Analysis

Statistical analyses and nonlinear regression were carried out with Graphad Prism 8.1.0. Statistical power calculations were made using Piface version 1.76.

## RESULTS

### Development of the ΔS-Cys-Alb Assay

#### 

##### Time and Temperature for the Ex Vivo Incubation

The highest temperature to which human P/S is exposed in its normal *in vivo* environment is 37 °C; as such, this temperature was chosen as the intentional *ex vivo* incubation temperature for the ΔS-Cys-Alb assay. To determine the time required to maximize S-Cys-Alb, as well as the impact of blood collection type and the effect of varying Cys and Cys-Cys concentrations on the time required to reach a maximum value of S-Cys-Alb, a matched collection of K_2_EDTA plasma, sodium heparin plasma, and serum from a healthy donor was obtained and S-Cys-Alb was measured in the freshly processed samples. Portions of each specimen were then fortified with an additional 1 μm Cys and 10 μm Cys-Cys or 2 μm Cys and 20 μm Cys-Cys. These added concentrations represent ∼1 and 2 standard deviations (SDs) of the mean values of ∼ 10 μm Cys and ∼ 62 μm Cys-Cys seen in the plasma of typical donors (20, 21). Nine-microliter aliquots of each unique P/S sample were then incubated in sealed 0.6-ml tubes at 37 °C and S-Cys-Alb was measured at 4, 18, 24 and 30-h time points. (Three separate 9-μl aliquots were made for each time point.) Differences between matched serum and plasma were negligible, with all specimens reaching their maximum value of S-Cys-Alb by 18 h. Addition of Cys and Cys-Cys to the samples resulted in a higher maximum value of S-Cys-Alb but did not alter the time required to reach it (supplemental Fig. S1).

##### ΔS-Cys-Alb Assay Precision, Linearity, Accuracy, Sensitivity, and Limits of Detection

As stated in the Introduction, we previously determined the inter-assay precision for measurements of S-Cys-Alb to be 1.6% (18). Because ΔS-Cys-Alb is the difference between two S-Cys-Alb measurements, the precision of ΔS-Cys-Alb can be calculated by propagating the error for this subtractive operation (23); when done so it is found to be 2.2%. Thus, for a ΔS-Cys-Albumin value of 20%, this corresponds to an inter-assay %CV of 11%.

The linearity of S-Cys-Alb measurements was determined by mixing known ratios of fully reduced and fully oxidized (S-cysteinylated) albumin in 10% increments from 0 to 100% S-Cys-Alb at a final concentration of 0.78 mm. Samples were then diluted 500-fold in 0.1% TFA and analyzed in technical replicates at each ratio (supplemental Fig. S2). The slope ± S.E., y-intercept ± S.E., and R^2^ value of the least-squares linear regression line were 1.0 ± 0.011, 0.76 ± 0.66, and 0.998, respectively. The slope of 1.0 indicates that the assay has unit sensitivity (*i.e.* change in instrument read-out per unit change in known relative concentration). Limits of detection were not determined as this figure of merit is not important to this assay given that > 99% of the U.S population has P/S albumin concentrations in the range of 490–810 μm (19). Likewise, there is no need for measurements of S-Cys-Alb in actual P/S that are < 5% or > 95% (see next section).

Overall accuracy was determined based on each data point from the aforementioned linearity experiment (supplemental Fig. S2). The average deviation from the known percent abundance of S-Cys-Alb was 0.78; the average absolute deviation was 1.2.

### Measurement of S-Cys-Alb and ΔS-Cys-Alb in Fresh Samples from Cardiac Patients

#### 

##### Population Estimates

**Fig. 2.**
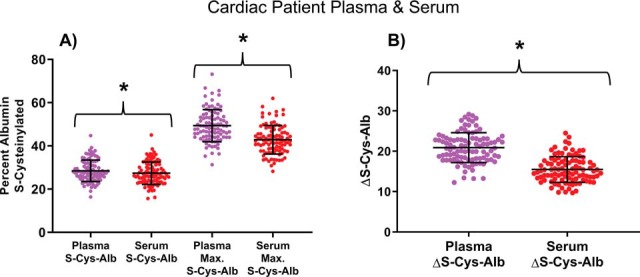
**S-Cys-Alb and ΔS-Cys-Alb in fresh, rapidly processed samples from cardiac patients.** *A*, S-Cys-Alb and maxed-out S-Cys-Alb observed in fresh samples from matched K_2_EDTA plasma and serum samples from cardiac patients undergoing coronary angiogram, cardiac stress test and/or coronary computed tomography angiography. *B*, ΔS-Cys-Alb, calculated from panel *A* by taking individual sample differences between maxed-out S-Cys-Alb and S-Cys-Alb. Error bars represent mean ± S.D.; *n* = 97 per group; * indicates a significant difference between means of indicated groups with *p* < 0.0001, paired t-tests.

Nonacute cardiac patients presenting with chest pain suggestive of coronary artery disease undergoing coronary angiogram, cardiac stress test and/or coronary computed tomography angiography at the recommendation of a cardiologist are likely to be individuals under continual low to moderate levels of systemic oxidative stress—a situation that could potentially raise their endogenous levels of S-Cys-Alb above that of nominally healthy individuals. As such, these cardiac patients represented a clinical population that could potentially pose a challenge to the theoretically usable dynamic range of the ΔS-Cys-Alb assay. To estimate the typical values of S-Cys-Alb and ΔS-Cys-Alb observed in fresh samples from these patients, matched K_2_EDTA plasma and serum samples were collected from 106 of them. P/S specimens were collected under rigorous guidelines (see Materials and Methods) to ensure the highest possible sample quality. Accordingly, samples from 9 patients were excluded because of hemolysis > 250 mg/dL (*n* = 7) or patient history of hemodialysis/kidney failure (*n* = 2; eGFR < 30 ml/min*1.73 m^2^). Fresh K_2_EDTA plasma and serum were found to have similar but significantly different values of S-Cys-Alb (paired *t* test, *p* < 0.001; Fig. 2). Following incubation of 9-μl aliquots at 37 °C for 18 h, S-Cys-Alb was measured again and the difference between the two measurements was recorded as ΔS-Cys-Alb. Both the maximum value of S-Cys-Alb and ΔS-Cys-Alb were found to be significantly higher in plasma than serum (paired *t* test, *p* < 0.001; Fig. 2). All distributions were Gaussian (D'Agostino and Pearson normality test; *p* > 0.05). The mean value of ΔS-Cys-Alb in cardiac patient plasma ± 95% CI of mean was 20.9% ± 0.75% and in serum was 15.5% ± 0.64%. Standard deviations were 3.7% and 3.2%, respectively. Empirically, these values suggest that ΔS-Cys-Alb values in 95% of fresh cardiac patient plasma and serum samples will fall in the ranges of 14–28% and 9.1–22%, respectively.

##### Time Courses

**Fig. 3.**
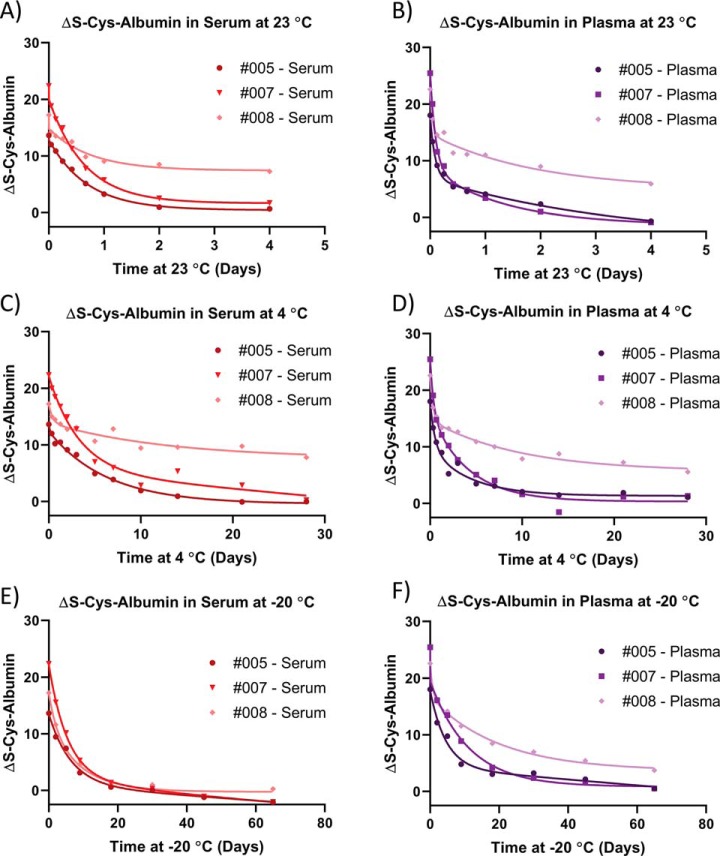
**Time courses for ΔS-Cys-Alb decay in matched serum and plasma from three separate patients (as indicated by patient numbers in the figure legends) at *A–B*, 23 °C, *C–D*, 4 °C, and *E–F*, −20 °C.** Lines drawn are meant to serves as visual guides.

Fifty-microliter aliquots of matched serum and plasma from three patients selected from each of the three tertiles of the population distributions for ΔS-Cys-Alb were incubated at 23 °C, 4 °C, and −20 °C for 4, 28, and 65 days, respectively (Fig. 3). Separate 50-μl aliquots were employed for each time point. Inter-individual variability in the (nonlinear) rates of decay of ΔS-Cys-Alb in both serum and plasma was evident at all temperatures.

### Quantitative Model for Ex vivo Formation of S-Cys-Alb

#### 

##### Determination of Rate Laws

The major reactions that govern the *ex vivo* formation of S-Cys-Alb in P/S include:

(1) $$AlbSH+Cys-Cys\overset{k_{1}}{\underset{k_{2}}{⇌}}SCysAlb+Cys$$

(2) $$2Cys+O_{2}\overset{Cu\left(II\right)}{\underset{k_{3}}{\to }}Cys-Cys+H_{2}O_{2}$$

where AlbSH is the native reduced form of albumin, Cys-Cys is cystine, SCysAlb is S-cysteinylated albumin, and Cys is cysteine.

The average initial starting concentrations of each reactant and product in P/S are known. As such, knowledge of the rate law governing the formation of S-Cys-Alb coupled with measurement of ΔS-Cys-Alb could, in theory, provide an estimate of the time spent by P/S samples at the temperature at which the rate law was determined. The rate law (including *k_3_*) for Eq. 2 was previously determined by Kachur *et al.* (26); the rate laws for the forward and reverse reactions in Eq. 1 were determined here at 23 °C.

**Fig. 4.**
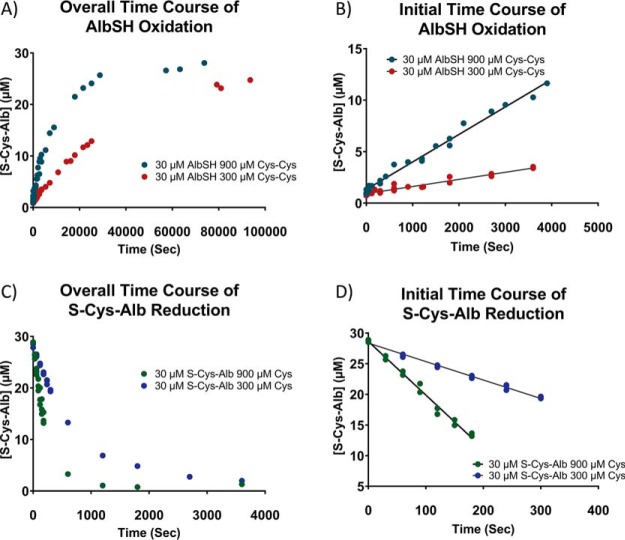
**Rates of albumin oxidation (S-cysteinylation) and reduction at 23 °C.** *A*, Overall time course for albumin S-cysteinylation (oxidation) and *B*, initial rate for albumin S-cysteinylation starting with 30 μm AlbSH and 300 or 900 μm Cys-Cys. *C*, Overall time course for reduction of S-Cys-Alb and *D*, initial rate for reduction of S-Cys-Alb starting with 30 μm S-Cys-Alb and 300 or 900 μm Cys.

**Fig. 5.**
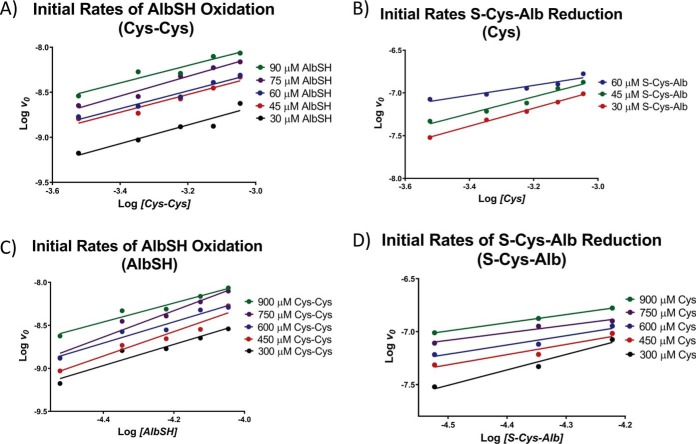
**Log-log plots employed to determine the reaction order for all species in the reversible oxidation (S-cysteinylation) of AlbSH by Cys-Cys (Eq. 1).** The average slopes for *A*, *B*, *C*, and *D* were 1.01 ± 0.05, 0.87 ± 0.25, 1.30 ± 0.17 and 0.96 ± 0.30, respectively. Thus, both the forward and reverse reactions were determined to be first order with respect to each reactant.

Eq. 1 is initially linear in both the forward and reverse directions (Fig. 4). Thus, to be able to model a simultaneous system of chemical reactions comprised of Eqs. 1 and 2, the forward and reverse rate laws for Eq. 1 were determined using the method of initial rates (27). To obtain albumin in fully reduced or fully oxidized form, high purity human serum albumin was fully reduced with Cys or fully oxidized with Cys-Cys, then spin-filtered to purify and concentrate the protein. Verification of complete reduction and oxidation was carried out by LC-MS analysis of the intact protein following alkylation with maleimide to verify that no structural disulfide bonds were reduced (supplemental Fig. S3). Starting conditions for all incubations employed to determine the forward and reverse rate laws for Eq. 1 are provided in supplemental Tables S1 and S2, respectively. The slopes of plots of log *v_o_* (initial rate) *versus* log [*reactant concentration*] (for several different co-reactant concentrations) revealed that the reaction order for all species in Eq. 1 was 1 (Fig. 5). Subsequently, the data in supplemental Table S1 were fit to the equation:

(3) $$v_{o}=k_{1}\left[AlbSH\right]\left[Cys-Cys\right]$$

(where *v_o_* is the initial reaction rate in M/s) and nonlinear regression was employed to determine that *k_1_* = 0.095 ± 0.017 m^−1^ s^−1^. Likewise, the data in supplemental Table S2 were fit to the equation

(4) $$v_{o}=k_{2}\left[SCysAlb\right]\left[Cys\right]$$

and nonlinear regression was employed to determine that *k_2_* = 3.37 ± 0.44 m^−1^ s^−1^. Given that *k_1_* and *k_2_* were determined at 23 °C, their values are consistent with the values of 0.6 ± 0.1 m^−1^ s^−1^ and 6.6 ± 0.4 m^−1^ s^−1^, respectively, that were recently determined by Bocedi *et al.* at 37 °C (28).

Once the forward and reverse rate laws for Eq. 1 had been determined, a series of differential equations that simultaneously consider the chemical reactions in Eqs. 1 and 2, and their respective rate laws, were assembled:

(5) $$\frac{d\left[AlbSH\right]}{dt}=-k_{1}\left[AlbSH\right]\left[Cys-Cys\right]+k_{2}\left[SCysAlb\right]\left[Cys\right]$$

(6) $$\frac{d\left[Cys-Cys\right]}{dt}=-k_{1}\left[AlbSH\right]\left[Cys-Cys\right]+k_{2}\left[SCysAlb\right]\left[Cys\right]+\frac{k_{3}\left[Cu\left(II\right)\right]\left[Cys\right]}{K_{z}\left(1+\frac{K_{y}}{\left[Cys\right]}\right)+\left[Cys\right]}$$

(7) $$\frac{d\left[SCysAlb\right]}{dt}=-k_{1}\left[AlbSH\right]\left[Cys-Cys\right]-k_{2}\left[SCysAlb\right]\left[Cys\right]$$

(8) $$\frac{d\left[Cys\right]}{dt}=k_{1}\left[AlbSH\right]\left[Cys-Cys\right]-k_{2}\left[SCysAlb\right]\left[Cys\right]-2\left(\frac{k_{3}\left[Cu\left(II\right)\right]\left[Cys\right]}{K_{z}\left(1+\frac{K_{y}}{\left[Cys\right]}\right)+\left[Cys\right]}\right)$$

In Equations 5–8, [Cu(II)] is the total concentration of copper (which stays constant), and *K_y_* and *K_z_* represent the first and second equilibrium dissociation constants pertaining to Cys complexation of Cu(II) that are involved in the copper-catalyzed oxidation of Cys to Cys-Cys at pH 7.4 as described by Kachur *et al.* (26). Eqs. 5–8 cannot be solved explicitly, but numerical solutions at any point in time are obtainable once all constants and starting concentrations are supplied.

##### Assessment of Quantitative Model in Serum and Plasma

To evaluate the ability of the combined rate law model (Eqs. 5–8) to predict formation of S-Cys-Alb in actual serum and plasma, matched serum and plasma were collected from a healthy donor. Serum was collected into and handled in trace metal-free tubes to facilitate quantification of copper. Initial concentrations of S-Cys-Alb, AlbSH, Cys-Cys and free and total copper for use in the predictive model were made as described in Materials and Methods. Measurement of Cys was deemed unnecessary because according to the model, by the time serum or plasma are processed from whole blood the concentration of Cys drops to a steady state at about 5 μm—regardless of whether or not the initial concentration starts at the low or high end of physiological Cys concentrations observed in human plasma (supplemental Fig. S4)—and which, in terms of Cys equivalents, is within the error of Cys-Cys quantification.

**Fig. 6.**
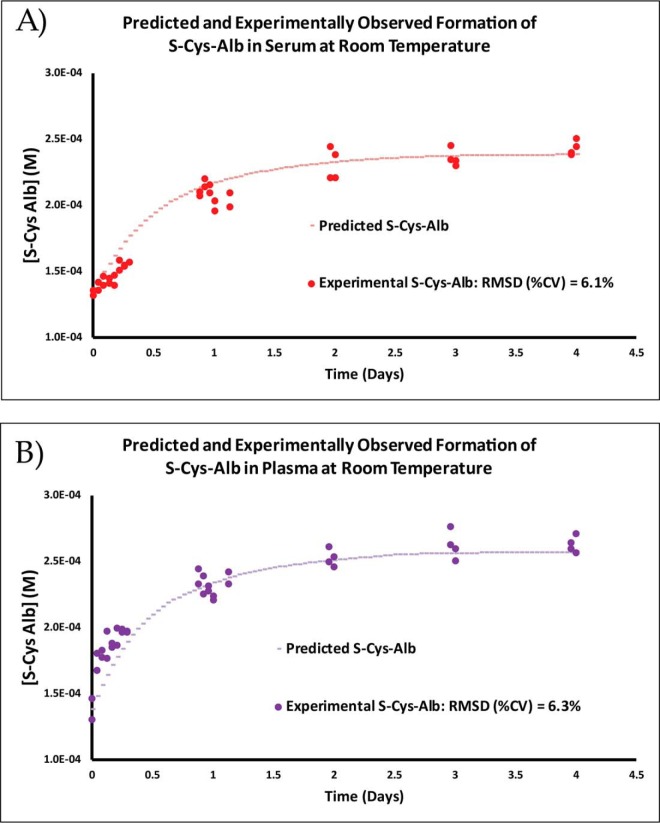
**Observed and rate law model-predicted formation of S-Cys-Alb in matched *A*, serum and *B*, K_2_EDTA plasma from a healthy donor.** Circles represent natural, unfortified serum or plasma containing initially measured concentrations of AlbSH = 609 μm (serum) or 605 μm (plasma); S-Cys-Alb = 134 μm (serum) or 138 μm (plasma); Cys-Cys = 52 μm (serum) or 58 μm (plasma); Cys = 5 μm (inferred, not measured, see “Results” text and supplemental Fig. S4); and Cu(II) = 12.6 μm. Dashed lines represent rate model-predicted trajectories for S-Cys-Alb formation based on numerical solutions to Eqs. 5–8 employing the rate and equilibrium constant parameters described in the main text.

Immediately following collection, aliquots for measurement of initial reactant and product concentrations were created and sent out for analysis or kept in-house at −80 °C until they were analyzed. A 100-μl aliquot of each specimen in a closed 1.5-ml snap-cap tube was then incubated at 23 °C for 4 days on a rotating vortex mixer (200 RPM), with numerous measurements of S-Cys-Alb collected initially and then at least once a day after Day 1. The data were then fit with the predictive model, using the empirically determined initial concentrations of all species along with rate and equilibrium constants pertinent to the model, determined as described above (*k_1_* and *k_2_*; Eq. 1 at 23 °C) or as previously determined (*k_3_*, *K_y_* and *K_z_*; Eq. 2 at 37 °C (26)) (Fig. 6). To best approximate the latter three parameters at the actual temperature of the experiment (23 °C), a value of −50 kJ/mol was estimated as the enthalpy of reaction per Cys ligand binding to Cu(II) based on the known enthalpy of Cys binding to other divalent cations (29) (the value for binding to Cu(II) is unknown). Integration of the van't Hoff equation provides the following formula to estimate the change in an equilibrium association constant (*K*) with temperature (*T*) given the estimated change in enthalpy (ΔH°) (30):

(9) $$\ln K_{2}=\ln K_{1}-\frac{\Delta H^{{}^{\circ}}}{R}\left(\frac{1}{T_{2}}-\frac{1}{T_{1}}\right)$$

where *R* is the ideal gas constant. Application of this formula to *K_y_* and *K_z_* resulted in a 2.5-fold decrease in each value (to 2.0 × 10^−6^ m and 3.5 × 10^−4^ m, respectively). The same factor was applied to estimate *k_3_* as 0.13 s^−1^ as well—a factor in the middle of the range of the fold-change expected for a 14 °C decrease in temperature (27)^2^. All plasma copper was assumed to be catalytically available and half of serum copper, 95% of which is bound to ceruloplasmin (33), was assumed to be catalytically available because only about 40% of ceruloplasmin-bound copper resides in the Cu(II) oxidation state (34). The RMSD fit (expressed as %CV) of the model to serum was 6.1% and that for K_2_EDTA plasma was 6.3% (Fig. 6). Models in which only free copper, only the ∼5% of copper not bound to ceruloplasmin, or all copper in serum were assumed to be catalytically available reveal that, as modeled, a major portion of bound copper must indeed be catalytically available (supplemental Fig. S5). Additional models in which *K_y_*, *K_z_*, and *k_3_* were run at their 37 °C-values and 10-fold below these values are also provided to illustrate how shifts in these parameters impact the kinetic model (supplemental Fig. S6).

Before initial freezing, separate aliquots of the matched serum and plasma samples were spiked with Cys and Cys-Cys (in a minimal volume of HBS buffer, pH 7.4) to increase the concentration of Cys by 12 μm and the concentration of Cys-Cys by 62 μm—putting these concentrations into a super-physiological range at twice the normal concentrations observed in normal human plasma (20, 21). Such concentrations of Cys and Cys-Cys have only been observed in patients with kidney failure (35). This experiment was conducted in order to test the hypothesis that oxygen could become rate-limiting under the extreme concentrations of Cys and Cys-Cys that can sometimes be observed in samples from patients with kidney failure. The observed albumin S-cysteinylation trajectory in the matched serum and plasma samples that were fortified with 62 μm Cys-Cys and 12 μm Cys did not match the model predictions in which oxygen is assumed to never become rate-limiting (supplemental Fig. S7). Taken together, the observed *versus* modeled results (Fig. 6 and supplemental Figs. S5–S7) reveal that a kinetics model in which oxygen is assumed to never become rate-limiting may be applicable to P/S samples with physiologically normal concentrations of Cys and Cys-Cys (Fig. 6), but that a new model that includes (depends on) [O_2_(*aq*)] as a reactant must be developed to accurately predict S-Cys-Alb formation kinetics in P/S samples from patients with kidney failure. Simulation results from a currently speculative model in which parameters for 1) the rate constant for re-oxidation of Cu(I) to Cu(II) by O_2_(*aq*) and 2) a constant that defines continual seepage of O_2_ into the reaction vessel are estimated and provided in supplemental Fig. S8. This model suggests that by accounting for [O_2_(*aq*)], it will likely be possible to use a *single* model to predict the kinetics of S-Cys-Alb formation in P/S from both physiologically normal patients and from renal failure patients that contain high concentrations of Cys and Cys-Cys.

**Fig. 7.**
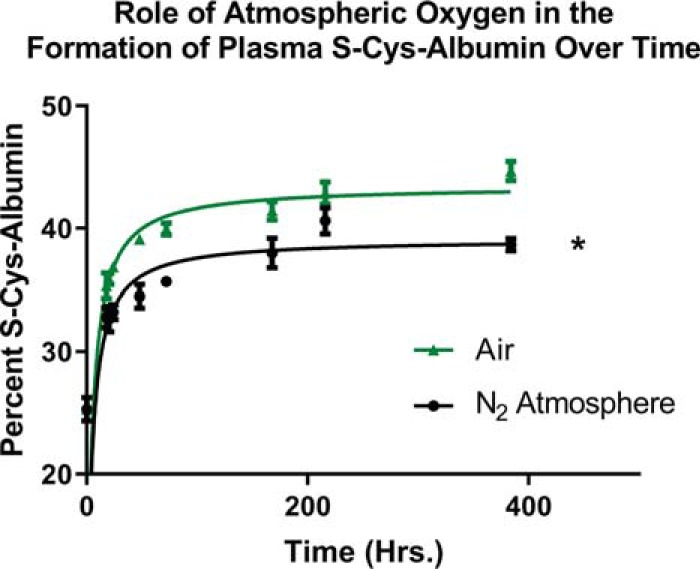
**Formation of S-Cys-Alb over time in aliquots of the same plasma incubated at 23 °C in air (green triangles) or under a nitrogen atmosphere (black circles).** The nitrogen atmosphere modestly but significantly lowered the total fraction of S-Cys-Alb formed (* *p* < 0.01; Wilcoxon matched pairs signed-rank test. *n* = 4 per time point; error bars represent S.D.)

To evaluate the practical effect on S-Cys-Alb of processing plasma normally then storing samples under an inert atmosphere in sealed vials, parallel incubations of freshly collected plasma in air and under a nitrogen atmosphere (using a Spilfyter “Hands-in-a-Bag” artificial atmospheric chamber) were conducted. Initial rates of S-Cys-Alb formation under the two atmospheres were nearly identical, but eventually diverged and resulted in a modestly lower maximum concentration of S-Cys-Alb in the sample incubated under nitrogen (Fig. 7). These experimental results compare favorably with the [O_2_(*aq*)]-dependent model in which a modest concentration of O_2_(*aq*) (70 μm) is assumed at the outset but no further O_2_ is allowed to enter the unfortified sample over time (supplemental Fig. S8, panel n, predicted *versus* observed unfortified samples).

### Blind Challenges of ΔS-Cys-Alb as a Marker of P/S Exposure to the Thawed State

Two separate challenges were conducted for ΔS-Cys-Alb as a marker of P/S exposure to thawed conditions in which the analyst was blinded to sample identities. The first study was a group-wise challenge wherein discrete groups of samples were either exposed to thawed conditions or properly stored at −80 °C *as groups*. The second blind challenge involved proper storage or mistreatment of discrete, individual samples. Before unblinding, the analyst was only aware that there would be a group-wise challenge and an individual sample challenge; he was unaware of any other aspect of the experimental design described below.

#### 

##### Group-wise Blind Challenge

**Fig. 8.**
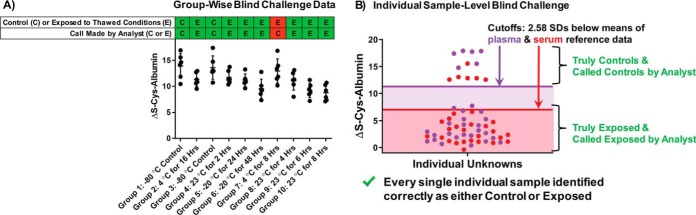
**Results from blinded challenges of the ability of ΔS-Cys-Alb to distinguish *A*, *groups* of samples exposed to various thawed conditions (listed below each group), and *B*, *individual* samples exposed to various thawed conditions including 23 °C for 24 h, 23 °C for 48 h, 23 °C for 72 h, 23 °C for 7 days, 4 °C for 7 days, 4 °C for 14 days, −20 °C for 60 days, −20 °C for 90 days (*n* = 6 per condition); controls kept at −80 °C (*n* = 12).** Out of a total of 70 calls of “control” *versus* “exposed” that had to be made between these two studies, only one was made incorrectly (see panel a).

Matched plasma and serum were collected from the same individual on three separate days, all spaced at least 6 days apart. This produced six unique but not highly disparate samples. Ten 50-μl aliquots were created from each sample, creating ten groups, each of which contained one aliquot each of the original six specimens. These groups were then randomly assigned: two groups were kept continually at −80 °C, and one group was subjected to each of the following eight conditions: 23 °C for 2 h, 23 °C for 4 h, 23 °C for 6 h, 23 °C for 8 h, 4 °C for 8 h, 4 °C for 16 h, −20 °C for 24 h, and −20 °C for 48 h. The samples were then given to the analyst with only a coded identifier on each sample that corresponded to its unique group. ΔS-Cys-Alb was then measured in each sample (Fig. 8*A*). The distributions of all data sets overlapped to some degree, indicating that it would likely be difficult to distinguish control group(s) from mistreated group(s). As such, data were analyzed using a statistical approach designed to limit type II errors (*i.e.* one-way ANOVA followed by uncorrected Fisher's LSD with *p* < 0.1 serving as the cutoff for statistical significance). Based on this analysis, it was clear that Groups 1 and 3 had higher mean values of ΔS-Cys-Alb than all other groups, except for Group 7, whose status was unclear. Moreover, the ΔS-Cys-Alb values in Groups 1 and 3 were consistent with fresh samples or those kept at −80 °C (cf. Fig. 2). Thus Groups 1 and 3 were named by the analyst as control groups. Group 7 could not be definitively categorized but was guessed/presumed to also be a properly handled control group. All other groups were assigned as having been exposed to thawed conditions of some sort. On unblinding it was revealed that all assignments except for Group 7 had been made correctly (Fig. 8*A*).

##### Individual Blind Challenge

Ten additional 50-μl aliquots were made from the six samples described in the preceding paragraph, producing a total of 60 specimens. These aliquots were made at the same time as the others to avoid an additional freeze-thaw cycle. Twelve of these specimens (*n* = 2 aliquots of each of the original six P/S samples) were kept at −80 °C. One aliquot each of the original six P/S samples was then subjected to each of the following eight conditions: 23 °C for 24 h, 23 °C for 48 h, 23 °C for 72 h, 23 °C for 7 days, 4 °C for 7 days, 4 °C for 14 days, −20 °C for 60 days, −20 °C for 90 days. The samples were then given to the analyst. Each individual test tube had only a single, completely unique coded identifier on it. ΔS-Cys-Alb was then measured in each sample. Because statistical analysis cannot be conducted on single samples, a ΔS-Cys-Alb integrity cutoff had to be assigned. Based on the Gaussian distribution of ΔS-Cys-Alb in plasma and serum from nominally unhealthy patients (Fig. 2), it can be predicted that 99% of fresh plasma samples from patients without renal failure have ΔS-Cys-Alb values in the range of 11–30%; and 99% of fresh serum samples have ΔS-Cys-Alb values in the range of 7.2–24%. These ranges were determined based on the means ± 2.58 SDs of the plasma and serum samples represented in Fig. 2*B*. Thus, ΔS-Cys-Alb values of 11% for plasma and 7.2% for serum were set as the cutoffs for this individual sample-level blind challenge. Using these cutoffs, all 60 individual specimens were categorized correctly (Fig. 8*B*).

### Case Study: Application of ΔS-Cys-Alb to Nominally Pristine Archived Serum Samples

**Fig. 9.**
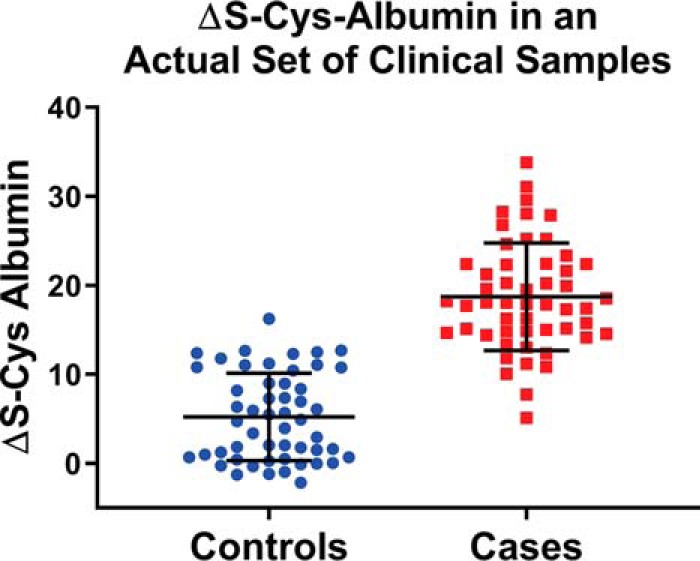
**ΔS-Cys-Alb results from a case study of serum samples with an excellent pedigree but in which an integrity discrepancy was suspected (see main text for details).** The values of ΔS-Cys-Alb in the controls barely overlapped with those of the cases (receiver operating characteristic curve c-statistic = 0.96) and the mean value of ΔS-Cys-Alb was strongly significantly lower in the controls than it was in the cases (*p* < 1 × 10–20; two-tailed student's *t* test). ΔS-Cys-Alb in the stage I lung cancer cases was essentially the same as it was in *fresh* samples from cancer free patients (cf. Figs. 2 and 8), meaning that the difference in ΔS-Cys-Alb between the cases and controls in this set cannot be because of the presence of cancer—leaving variable exposure to the thawed state as the only reasonable explanation for the difference observed. This was subsequently confirmed by the sample providers.

**Fig. 10.**
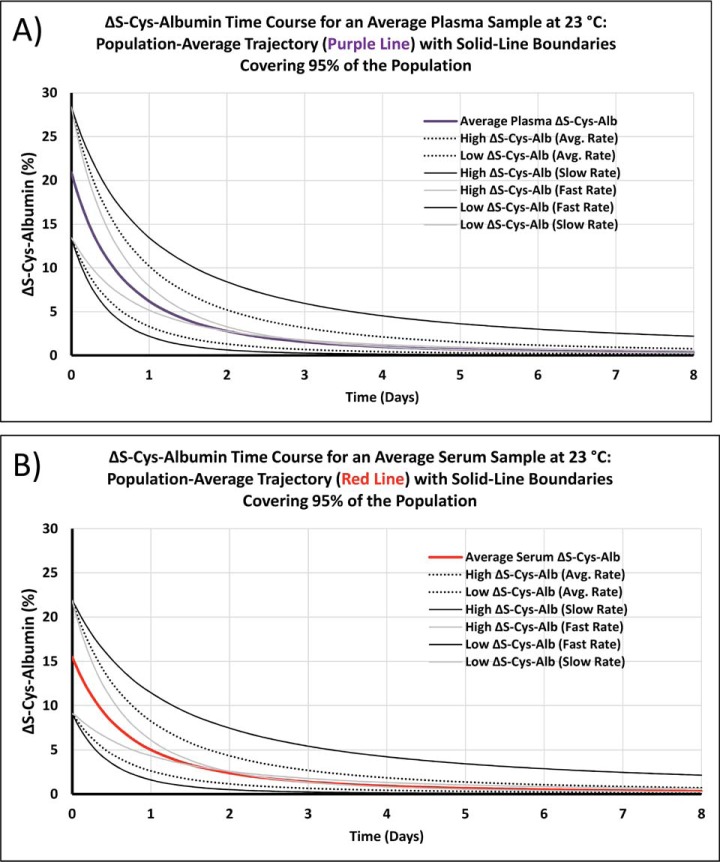
**Modeled time course trajectories for ΔS-Cys-Alb in *A*, plasma and *B*, serum.** Colored curves indicate average trajectories based on known population average starting concentrations for all relevant reactants, products and the copper catalyst. Rate model constants are the same as those used to create Fig. 6 and are given in the main text. Curves with average rates (including the colored traces) are based on the population average values for P/S copper (58) and the ratio of AlbSH/S-Cys-Alb determined here (Fig. 2). Curves with fast rates or slow rates are based on P/S copper concentrations and AlbSH/S-Cys-Alb ratios that are 2 SDs above or below the population averages. All starting reactant, product and catalyst concentrations are provided in supplemental Table S3. These curves are useful for relating ΔS-Cys-Alb measurements in unknown samples to the amount of time the sample(s) have spent at the equivalent of 23 °C: For *groups* of unknown samples, the measured ΔS-Cys-Alb mean ± 95% CI can be overlaid on the colored traces to estimate the mean ± least and greatest amounts of exposure time. For *individual* unknown samples, the measured ΔS-Cys-Alb value can be related to exposure time via the colored trace and the least and greatest likely exposure times can be estimated from the solid black lines.

Following development of the ΔS-Cys-Alb assay, an unplanned event occurred in our laboratory in which it was needed. In short, a set of serum samples from stage I lung cancer patients and corresponding age, gender and smoking-status matched controls were undergoing glycan “node” analysis (36–40) as part of an unrelated project. The samples were collected under NIH-sponsorship by seasoned investigators with well-defined standard operating procedures and, on paper, there should not have been any specimen integrity problems. As part of the glycan “node” assay, relative blood glucose concentrations were (unintentionally) determined. The relative blood glucose concentrations in these samples indicated unexpectedly elevated levels of blood glucose in the cases. We have previously observed that albumin glycation increases significantly over time in P/S samples exposed to thawed conditions (41); as such, despite the fact that there was a pristine paper trail associated with these samples, we decided to measure ΔS-Cys-Alb in them. The mean values of ΔS-Cys-Alb were significantly different between the cases and controls (*p* < 1 × 10^−20^; student's *t* test) and there was nearly no overlap in their ΔS-Cys-Alb distributions (Fig. 9). Because ΔS-Cys-Alb is not a marker of stage I lung cancer, these data indicated an integrity discrepancy between the two sets of serum samples. On showing these data to the clinical investigators who had provided the samples, it was ultimately disclosed that the −80 °C freezers in which the control samples had been stored had experienced a power outage for about 3–4 days during a natural disaster. Using the combined rate law model described above (Eqs. 5–8) in combination with the average population values for ΔS-Cys-Alb determined here (Fig. 2), the trajectories of ΔS-Cys-Alb in fresh plasma and serum samples with low, average and high ΔS-Cys-Alb values running at low and high rates of reaction (where rates are largely dependent on P/S copper concentration) were calculated (Fig. 10). From the average ΔS-Cys-Alb with average decay rate curve for serum (red line in Fig. 10*B*) and the mean ± 95% CI of 5.2 ± 1.4 from the control set of samples from this lung cancer study (Fig. 9), it was estimated that the average control serum samples had been exposed to the equivalent of room temperature (∼ 23 °C) for 23 h with lower and upper 95% CI-based bounds of 17 and 32 h—an estimate that aligns with the fact that despite losing power for 3–4 days, the freezers had not been opened.

## DISCUSSION

### 

#### 

##### Utility of ΔS-Cys-Alb

ΔS-Cys-Alb serves as an effective indicator of P/S exposure to thawed conditions (temperatures > −30 °C (10, 11)) over time frames at 23 °C, 4 °C, and −20 °C (Fig. 3) that are well aligned with the stability of many important clinical analytes (42–44). As an endogenous marker that serves as a P/S QA tool, it is unique in that not only is its mechanism of formation known, but the chemical rate law governing its change in P/S over time has also been established. This level of characterization of an endogenous P/S QA marker is without precedent.

As illustrated in the blind challenge set of experiments, ΔS-Cys-Alb, in practice, may be measured and interpreted within questionable *group*(*s*) of samples or in *individual* samples that have no connection to any other samples whatsoever. In doing so, a single *group* may be statistically compared with the population mean of ΔS-Cys-Alb in fresh plasma or serum as established here. Multiple groups may be compared with one another (*e.g.* as in Fig. 8*A*) or to the aforementioned population means. The number of samples required from a *group* of plasma or serum samples to achieve at least 80% power to detect a particular exposure time at the equivalent of room temperature are provided in Table I. This table should serve as a useful guide with regard to planning QA/QC inquiries into existing plasma or serum sample sets. *Individual* samples, on the other hand, must be compared with a population distribution-based ΔS-Cys-Alb cutoff value—such as those 2.58 SDs below the population means established for plasma and serum in the individual-level blind challenge here.

**Table I:** ΔS-Cys-Albumin kinetics for average plasma and serum samples at 23 °C, with the corresponding number of samples required (n-values; based on statistical power calculations) to detect the indicated time of exposure^a^

Time at 23 °C (Hrs)	Plasma		Serum	
	ΔS-Cys-Albumin	One-Group Comparison to Population Mean at Time 0: n for ≥ 80% Power to Detect Exposure Time^b^	ΔS-Cys-Albumin	One-Group Comparison to Population Mean at Time 0: *n* for ≥ 80% Power to Detect Exposure Time^b^
0	20.9	–	15.5	–
1	19.6	52	14.6	79
2	18.4	15	13.8	23
3	17.3	8	13.0	12
4	16.3	6	12.3	8
5	15.4	5	11.7	6
6	14.5	4	11.1	5
7	13.7	4	10.5	5
8	13.0	4	10.0	4
9	12.3	3	9.5	4
10	11.7	≤ 3	9.0	4
11	11.1	≤ 3	8.6	4
12	10.6	≤ 3	8.2	3
13	10.1	≤ 3	7.9	≤ 3
14	9.6	≤ 3	7.5	≤ 3
15	9.1	≤ 3	7.2	≤ 3
16	8.7	≤ 3	6.9	≤ 3
17	8.3	≤ 3	6.6	≤ 3
18	8.0	≤ 3	6.3	≤ 3
19	7.7	≤ 3	6.1	≤ 3
20	7.3	≤ 3	5.8	≤ 3
21	7.0	≤ 3	5.6	≤ 3
22	6.7	≤ 3	5.4	≤ 3
23	6.5	≤ 3	5.2	≤ 3
24	6.2	≤ 3	5.0	≤ 3

^a^Initial concentrations and other parameters used in the kinetics model are provided in supplemental Table S3. Plots of these time courses up to 8 days are in Fig. 10.

^b^One-sample (group), one-tailed t-test; α = 0.05; σ_Plasma_ = 3.71; σ_Serum_ = 3.19.

##### Alignment with Theoretical Predictions

In most fresh samples, ΔS-Cys-Alb in plasma and serum is above 10% and 12%, respectively, (Fig. 2*B*). The observed range of ΔS-Cys-Alb in plasma and serum (Fig. 2*B*) lies in-line within the theoretical range of 11–38% for ΔS-Cys-Alb that can be predicted based on the average plasma concentrations of albumin, Cys-Cys and Cys observed in the human population (19–21). This predicted range of ΔS-Cys-Alb does not consider the possibility that other P/S proteins, such as alpha-1-antitrypsin may consume Cys-Cys/Cys equivalents in thawed P/S (45)—potentially accounting for slightly lower mean values of ΔS-Cys-Alb than predicted in both plasma and serum. Notably, the overall degree of inter-individual variability observed would be expected for any endogenous marker of P/S integrity.

Besides inter-individual differences in ΔS-Cys-Alb, there are inter-individual differences in the kinetics of ΔS-Cys-Alb decay over time at temperatures above the freezing point of P/S (Fig. 3). Predictions for the inter-individual variability in time course trajectories at 23 °C were provided (Fig. 10). These are in line with the variability in person-to-person trajectories observed in Fig. 3. Notably, the time course trajectory for serum sample #008 tends to float above the other two samples at 23 °C and 4 °C but is aligned with the other two samples at −20 °C (Fig. 3). This did not occur for the plasma samples. As we have not yet determined the rate law at −20 °C this observation opens up a new question about the behavior of ΔS-Cys-Albumin in serum as it approaches its freezing point. At this point we do not yet have an evidence-backed explanation for this phenomenon. Most likely it is related to the facts that (1) plasma and serum do not exhibit simple eutectic behavior near their freezing point of −30 °C (46, 47) and (2) the seemingly unique properties of plasma and serum from this particular patient are mitigated by this noneutectic behavior in serum but not plasma. This was an unexpected observation, but it does not contradict other data presented herein or diminish the functional utility of the ΔS-Cys-Alb assay with regard to its ability to detect minor exposures to thawed conditions within small *groups* of samples (Fig. 8*A*) or longer exposures in *individual* samples (Fig. 8*B*); moreover, the potential variability within the time course trajectory predictions (kinetics) is well-estimated (Fig. 10). As such, given the potentially open-ended nature of an inquiry to explain this phenomenon, it will be investigated in a future study.

##### ΔS-Cys-Alb Differences Between Plasma and Serum

Matched plasma and serum samples were prepared (diluted) and run interspersed with one another on the LC-MS instrument—with the same analyst preparing both the plasma and serum samples. As such, systematic error is not likely to account for the observed difference between plasma and serum. The major source of the discrepancy in ΔS-Cys-Alb between matched plasma and serum samples was not the initial value of S-Cys-Alb but was rather the maximum value reached following incubation for 18 h at 37 °C (Fig. 2*A*). This discrepancy is not observed in all samples; yet the source of the discrepancy is unclear as it is not related to the *difference* in pre-centrifugation delay between the matched serum and plasma samples, nor is it related to serum clotting time (supplemental Fig. S9). Neither is it related to the visually documented degree of hemolysis or the *difference* in degree of hemolysis between plasma and serum (hemolysis data not shown). Clotting factors such as Factor XIII contain free Cys residues and may consume some free Cys and/or Cys-Cys equivalents during the clotting process—which in some, but not necessarily all cases (supplemental Fig. S9), may consume a significant portion of available free Cys and/or Cys-Cys equivalents. This would make these equivalents unavailable for reaction with albumin during serum storage.

Matrix effects cannot yet be ruled out as a contributing explanation to the differences in ΔS-Cys-Alb between matched plasma and serum. These are unlikely to play a substantial role, however, because they would depend on there being some compositional difference between plasma and serum that *varies in magnitude between individuals* (reaching zero in some cases—see supplemental Fig. S9) and *preferentially* suppresses either the native or S-cysteinylated proteoform of albumin to a greater extent than the other form whereas both forms are present in P/S at relatively similar absolute concentrations. Moreover, nearly all of the difference is carried by the second measurement of S-Cys-Alb (Fig. 2*A*)—which would not be expected if matrix effects alone were responsible for the difference.

Given that the existence of a difference between plasma and serum does not impact the functional utility of the ΔS-Cys-Alb assay toward detection of samples that have been exposed to thawed conditions (Figs. 8–9) and that identification of the source(s) of the difference is currently an open-ended problem, identification of the source(s) will be addressed in a future study.

##### Importance and Limitations of the Rate Law

The chemical reactions that contribute to S-Cys-Alb formation in P/S *ex vivo* are known (Eqs. 1–2). This made it possible to move beyond empirical cataloguing of instability trends and actually determine rate laws and develop a mathematical model to facilitate (1) prediction of how the QA marker will behave across a wide range of reactant and product starting concentrations and (2) back-calculation of the approximate time at which a P/S specimen has been exposed to the temperature at which the rate law was determined (Fig. 10).

The initial rate law model developed here assumes that the concentration of dissolved oxygen, [O_2_(*aq*)], in P/S is not rate-limiting. This appeared to hold true in samples that possessed a physiologically normal concentration of Cys and Cys-Cys (Fig. 6). Two observations, however, suggested that the [O_2_(*aq*)] in P/S was very close to becoming rate-limiting: First, storage of unfortified plasma under nitrogen after initial processing was found to limit the overall formation of S-Cys-Alb (Fig. 7). And second, the rate at which S-Cys-Alb forms in unfortified P/S samples was accurately predicted by the model that does not take [O_2_(*aq*)] into account whereas the rate at which S-Cys-Alb forms in P/S samples fortified with extra Cys and Cys-Cys is significantly overestimated by this model (supplemental Fig. S7). Yet the rate at which S-Cys-Alb forms in both unfortified and fortified P/S can be predicted using a model that takes [O_2_(*aq*)] into account (supplemental Fig. S8). Thus, the present model based on Eqs. 5–8 alone, should be employed only under two conditions: First, when P/S samples are known to have been stored under air, and second, in patient populations without kidney disease requiring hemodialysis.

The rate and equilibrium constants associated with Eq. 2 were determined for Cu(II) in a 40 mm sodium phosphate buffer (26)—a solution wherein the copper ions would be complexed to the various protonated forms of phosphate ions present. In serum, however, 95% of copper is bound to ceruloplasmin (33) and in K_2_EDTA plasma essentially all of the copper is bound to the ∼ 5 mm EDTA present. As such, the values of *k_3_*, *K_y_* and *K_z_* employed here—although shown to be reasonably accurate empirically (Fig. 6; supplemental Fig. S6)—are not necessarily accurate representations of these values as they exist in P/S. This may explain why the predicted rates of S-Cys-Alb formation in serum and plasma are, for the first several hours, faster and slower, respectively, than observed (Fig. 6). Transition metals besides copper—most prominently iron—may also play some role in catalyzing Eq. 2 in P/S. “Free” or nontransferrin bound iron is typically in the nm range in serum (48). Moreover, we have previously observed that Cu(II) ions are a far more efficient catalyst of intramolecular disulfide bond formation than are Fe(III) ions (49). As such, “free” iron in serum likely contributes negligibly to Eq. 2. In plasma, however, Fe(III)-EDTA may play a significant role in this reaction—potentially accounting, at least in part, for the higher-than-predicted initial rate of S-Cys-Alb formation (Fig. 6). Efforts are underway to develop a comprehensive rate law that takes [O_2_(*aq*)] and all relevant metals, complexed as they are within P/S, into account across the range of temperatures likely to be encountered by P/S samples.

##### Practical Impact of Storing P/S Under an Inert Atmosphere

We have previously shown that the degree of air headspace above P/S samples stored at −20 °C does not significantly impact the overall rate of S-Cys-Alb formation (18). However, dissolved oxygen is involved in the oxidation of Cys to Cys-Cys (Eq. 2) and its potential range in an aqueous solution such as P/S (0–250 μm (16, 17)) lies in the range of the total concentration of Cys equivalents in P/S—which includes the Cys equivalents in Cys-Cys and can exceed 150 μm. As such, we evaluated the impact of nitrogen as a headspace gas (relative to air) on the formation profile of S-Cys-Alb in plasma. The results (Fig. 7) suggest that once P/S samples are exposed to air, storing them under an inert atmosphere may provide a modest but significant ability to mitigate oxidative biomolecular damage.

##### Implications of Study Results for Single Measurements of S-Cys-Alb

Several studies have suggested that S-Cys-Alb may be useful as a marker of systemic oxidative stress in various disease conditions (50–53). As shown here, S-Cys-Alb's susceptibility to change *ex vivo* undermines the utilization of S-Cys-Alb as a biomarker of oxidative stress unless *extreme* care is taken to *rigorously* document specimen exposure to thawed conditions before measurement. At the same time, the fact that the range of S-Cys-Alb observed in fresh samples overlaps with the range of maximum values of S-Cys-Alb observed after samples have been intentionally incubated in the thawed state (Fig. 2*A*) precludes the use of a single measurement of S-Cys-Alb as a marker of P/S integrity.

##### Limitations of ΔS-Cys-Alb

Two known confounders place minor limits on the use of ΔS-Cys-Alb as a P/S QA tool: First, patients with poor kidney function who require hemodialysis are susceptible to abnormally elevated levels of circulating Cys and Cys-Cys (35); they may also have elevated levels of S-Cys-Alb (51, 54–56)—though these studies did not explicitly consider the possible *ex vivo* formation of S-Cys-Alb. Elevated S-Cys-Alb *in vivo* does not impact ΔS-Cys-Alb, but elevated circulating Cys and Cys-Cys may account for ΔS-Cys-Alb levels above 40%. Such samples would take slightly longer periods of time to reach the lower values of ΔS-Cys-Alb considered to represent samples exposed to thawed conditions. However, if the samples are known to come from kidney failure patients, this fact can potentially be considered vis-à-vis the rate law established above. Second, human albumin mutations represent the only qualitative ΔS-Cys-Alb assay confounder. These are rare in most populations (with average rates of 0.001–0.03% (57))—but even if such samples were measured, the highly accurate mass spectrometric measurements of the intact protein on which the ΔS-Cys-Alb assay is based would detect all but the inconsequential isobaric protein variants.

##### Linking ΔS-Cys-Alb to the Stability of Clinically Important Biomolecules

The degree to which the measurable quantities of other biomolecules, including unrelated proteins/proteoforms, lipids, and nucleic acids are impacted during the time it takes ΔS-Cys-Alb to reach zero will be described in separate publications. A few articles in the past several years have tabulated the stabilities of common clinical analytes in P/S, many of which are unstable within time frames that would readily be detected by ΔS-Cys-Alb (42–44). Such co-instability with ΔS-Cys-Alb illustrates that ΔS-Cys-Alb does not change too rapidly or too slowly to possess substantial QA/QC utility. Conceptually, we view the development of a low-volume, inexpensive P/S QA marker that is based on known chemical reactions and their established rate laws (*i.e.* mathematical model) that can be used to approximate the amount of time a specimen has spent at the equivalent of room temperature to represent a critical waypoint in biobanking quality assurance. Ultimately, however, setting a ΔS-Cys-Alb cutoff that defines samples as “bad” depends on the intended use(s) of the samples. Moreover, prioritizing the tradeoff between keeping/using “bad” samples and throwing away “good” samples will always involve economic as well as scientific considerations. This means that functionally clarifying the meaning of QA marker measurements will always be context-dependent. Yet because the kinetic behavior of ΔS-Cys-Alb has been well defined here (at least at 23 °C), the only “added ingredient” necessary to link clinically important biomarkers of interest to ΔS-Cys-Alb is to *independently* characterize their empirical stability in P/S at room temperature, which is often done as part of careful analytical method development. Once this has been done, the time point at which initial instability occurred can be mapped to a clinical marker-specific ΔS-Cys-Alb cutoff vis-à-vis the kinetics model established here (see, for example, Fig. 10). This will allow the rapid, inexpensive, low-volume measurement of ΔS-Cys-Alb in unknown samples to provide direct insights into the validity of clinical biomarker measurements in any archived P/S specimen, regardless of their presumed storage and handling history.

## CONCLUSIONS

ΔS-Cys-Albumin, measured via a low-volume (≤10 μl), dilute-and-shoot, LC/MS assay, is an effective protein oxidation-based QC/QA marker for plasma and serum exposure to thawed conditions (*i.e.* > −30 °C). Both the mechanism of *ex vivo* change for ΔS-Cys-Albumin and its rate law have been established. Though in need of fine tuning vis-à-vis determination of the rate law for oxidation of cysteine to cystine under conditions highly specific to *ex vivo* plasma and serum, the multireaction rate law governing the formation of S-Cys-Albumin has been determined and empirically shown to be capable of accurately predicting S-Cys-Albumin formation in plasma and serum. Thus, when population averages of the relevant reactants are assumed, the combined rate law facilitates estimation of the time frame over which plasma and serum samples with unknown storage and handling histories have been exposed to the equivalent of room temperature conditions. As such, the stability of any clinical biomarker of interest can readily be linked to ΔS-Cys-Albumin by conducting a room temperature stability study of the marker—regardless of the mechanism by which it exhibits instability. When plasma and serum samples are grouped, we have shown that ΔS-Cys-Albumin can detect room temperature exposures of as little as 2 h—yet several days at room temperature are required for plasma and serum samples to reach the ΔS-Cys-Albumin minimum value of zero. By modeling the rate law using the population survey of fresh, matched plasma and serum from cardiac patients as input data, it can be seen that as few as 12 serum or 8 plasma samples from an unknown group are required to provide ≥ 80% power to detect 3 h of exposure to the equivalent of room temperature conditions. Mistreatment of individual samples can also be detected when their ΔS-Cys-Albumin values are 3 SDs below the population means (*i.e.* < 11% for plasma or 7.2% for serum). In summary, ΔS-Cys-Albumin readily identifies poorly stored or handled plasma and serum specimens and provides investigators a robust tool by which to prevent their inclusion in clinical research studies.

## Supplementary Material

supplemental Fig. S4

Supplemental Data

## Footnotes

^2^ In a simple system consisting of only buffer, Cu^2+^, and Cys, Eq. 2 exhibits two different kinetics phases—the first of which is faster and runs until, nominally, the molar concentration of Cys is equal to that of Cu^2+^ (26, 31). In the absence of a second co-reaction (i.e., Eq. 1) that generates fresh free thiol-containing Cys, all Cys is then bound to copper; hydrogen peroxide then accumulates in the absence of free Cys and subsequently drives the second reaction phase, the rate constant for which, at 37 °C, is 4.25-fold lower than the rate constant of the first phase (26). In P/S, Eq. 1 continually generates fresh Cys; moreover, the catalase concentration in P/S is about 50 U/mL (32)—a quantity sufficient to prevent accumulation of hydrogen peroxide and therefore the second reaction phase (26)—as such, only the rate constant for phase 1 of Eq. 2 was considered in the models presented here.

^1^ The abbreviations used are:

P/S: plasma and serum
PTMs: post-translational modifications
Cys: cysteine
Cys-Cys: cystine
S-Cys-Alb: S-cysteinylated albumin
QC: quality control
QA: quality assurance
SD: standard deviation
SE: standard error.
