# Supplementary material for: Delta-S-Cys-Albumin: A Lab Test that Quantifies Cumulative Exposure of Archived Human Blood Plasma and Serum Samples to Thawed Conditions
Source: Mol Cell Proteomics. 2019 Jul 19;18(10):2121–37. doi: 10.1074/mcp.TIR119.001659 (PMC6773563; doi:10.1074/mcp.TIR119.001659)
Supplement: supplemental Fig. S4 [file TIR119.001659_index.html]

Supplement to Delta-S-Cys-Albumin: A Lab Test that Quantifies Cumulative Exposure of Archived Human Blood Plasma and Serum Samples to Thawed Conditions | Molecular & Cellular Proteomics

## Supplemental Data

- Supplemental Data - Supplemental Figures and Tables with references cited therein.
