# Supplementary material for: Delta-S-Cys-Albumin: A Lab Test that Quantifies Cumulative Exposure of Archived Human Blood Plasma and Serum Samples to Thawed Conditions
Source: Mol Cell Proteomics. 2019 Jul 19;18(10):2121–37. doi: 10.1074/mcp.TIR119.001659 (PMC6773563; doi:10.1074/mcp.TIR119.001659)
Supplement: Supplemental Data [file 154084_0_supp_357197_pv15cr.pdf]

Supplemental Data

Delta-S-Cys-Albumin: A Lab Test that Quantifies Cumulative Exposure of Archived Human Blood Plasma & Serum Samples to Thawed Conditions

Running Title: ΔS-Cys-Albumin: A Marker of Plasma/Serum Integrity

Joshua W. Jeffs [a], Nilojan Jehanathan [a], Stephanie M. F. Thibert [a], Shadi Ferdosi [a], Linda Pham [b], Zachary T. Wilson [b, c], Christian Breburda [b, c], and Chad R. Borges [a, *]

Supplemental Data Includes

Detailed Methods

Figures S1-S9

Tables S1-S3

Supplemental Data References

DETAILED METHODS

Materials/Reagents

Highly purified human serum albumin (Cat. No. A3782), L-cysteine (C7352), trifluoroacetic acid (TFA, 299537), L-Cystine (30200), L-Cystine dihydrochloride (C2526), L-Cysteine-2,3,3,-D3 (701424), Amicon Ultra-4 centrifugal filter, MWCO = 50 kDa (Z740191), ammonium hydroxide (320145), ammonium bicarbonate (A6141), sodium sulfate (S9627), deferoxamine mesylate salt (D9533), HEPES (H3375) and NaCl (S7653) for preparation of HEPES-buffered saline solution (HBS buffer) were purchased from Sigma-Aldrich (St. Louis, MO). LC-MS grade Acetonitrile (TS-51101), LC-MS grade water (TS-51140), hydrochloric acid (50-878-166), Pierce BCA protein assay kit (23227), and methanol (A456) were purchased from ThermoFisher Scientific (Waltham, MA). Chelex 100 resin (1421253) was purchased from Bio-Rad Laboratories (Hercules, CA). SPE cartridges (SPE-P0005-03BB) purchased from Silicycle (Quebec, Canada). L-Cystine-3,3,3',3'-D4 (DLM-9812-PK) was purchased from Cambridge Isotope Laboratories, Inc. (Andover, MA). N-Methyl-N-Trimethylsilyl Trifluoroacetamide (MSTFA) (24589-78-4) was purchased from Regis Technologies (Morten Grove, IL). All non-LC-MS solvents were of HPLC grade.

LC-ESI-MS

The data reported here were collected on different instruments using a modified HPLC step-gradient that provided nearly complete chromatographic separation of albumin and apolipoprotein A-I. Relative quantification of intact albumin proteoforms was done by liquid chromatography-electrospray ionization-mass spectrometry (LC-ESI-MS) on either a Dionex Ultimate 3000 HPLC equipped with a 1:100 flow splitter connected to a Bruker maXis 4G

quadrupole-time-of-flight (Q-TOF) mass spectrometer or an Agilent 1260 Infinity II HPLC connected to an Agilent 6530 ESI-Q-TOF instrument. Both instruments have an ion source design in which the spray needle is held at ground and the inlet of the instrument is brought to a high negative potential in positive ion mode. The Agilent instrument was employed for time course measurements at different temperatures, linearity evaluation, and for ~ 2/3 of the ΔS-Cys-Alb population measurements; the Bruker instrument was used for everything else. In the following instrument and data processing descriptions, Agilent instrument parameters are listed in brackets next to the Bruker instrument parameters: A trap-and-elute form of LC-MS was carried out in which 5 μL [10 μL] of sample was loaded via a loading pump at 10 μl/min [200 μl/min] in 80% water containing 0.1% formic acid (Solvent A) / 20% acetonitrile [containing 0.1% formic acid] (Solvent B) onto an Optimize Technologies protein captrap configured for uni-directional [bi-directional] flow on a 10-port diverter valve. The trap was then rinsed at this solvent composition with the HPLC loading pump at 10 μl/min [200 μl/min] for 3 minutes [1 minute]. The flow over the captrap was then switched to the micro pump, which was set at a flow rate of 3 μL/min [200 μl/min] and composition of 65/35 A/B. This composition was held until 4.5 min [2.5 min.]. From 4.5-4.6 min. [2.5-2.6 min.] the composition was ramped to 55/45 A/B then held. From 7.5-7.6 min. [5.5-5.6 min.] the composition was ramped to 20/80 A/B then held. Then from 9.4-9.5 min.[6.6-6.7 min.] the composition was ramped back to 80/20 A/B in preparation for the next injection. Following the valve switch at 3 minutes [1 minute], the captrap eluate was directed to the mass spectrometer operating in positive ion, TOF-only mode, acquiring spectra in the m/z range of 300 to 3000 [100 – 3,200]. ESI settings for the Agilent G1385A [dual AJS ESI] capillary microflow nebulizer ion source were as follows: End Plate Offset -500 V, Capillary -4500 V [VCap 5,700 V;

nozzle voltage (expt) 2,000 V], Nebulizer nitrogen 3 Bar [45 psig], Dry Gas nitrogen 3.0 L/min at 225 °C [7 L/min at 325 °C; sheath gas 11 L/min at 250 °C]. Data were acquired in profile mode at a digitizer sampling rate of 4 GHz. Spectra rate control was by summation at 1 Hz.

Data Analysis: As previously described (1), approximately 1 minute of recorded spectra were averaged across the chromatographic peak apex of albumin. The electrospray ionization charge-state envelope was deconvoluted with Bruker DataAnalysis v4.2 software [MaxEnt software] to a mass range of 1000 Da on either side of any identified peak. Charge deconvolution settings were established to ensure that the relative peak widths and signal-to-noise ratios of the raw spectra were reproduced in the deconvoluted spectra. Deconvoluted spectra were baseline subtracted to the same degree on both instruments, and all peak heights were calculated, tabulated and exported to a spreadsheet for further analysis. Peak heights were used for quantification as opposed to peak areas, because of the lack of baseline resolution for some of the peaks.

Rate Law Determination

The approach to rate law determination entailed measuring the initial rate of the forward (and, separately, reverse) reaction by plotting S-Cys-Alb (or AlbSH) concentration in molar units (M) vs. time (s). The initial phase of these plots was linear. From this initial linear phase, the slope was obtained and recorded as the initial rate (v_0). This process was repeated for multiple starting concentrations of reactants in which none of the products were initially present.

This latter requirement made it necessary to create and isolate AlbSH in which no S-Cys-Alb was present and, for the reverse reaction, S-Cys-Alb in which essentially no AlbSH was

present. Pure AlbSH was prepared starting with a 1 mM concentration of commercially prepared albumin in HBS buffer (pH 7.4). The sample was aliquoted into 500 μL volumes and mixed with 300 μL of 10 mM Cys, followed by incubation for 1 hour at room temperature. After the incubation period, 1 μL of sample was diluted in 600 μL of 0.1% TFA and analyzed to verify complete reduction of albumin. The aliquots were then split into 200 μL aliquots in Amicon Ultra-4 centrifugal spin filters (MWCO 50K). Each sample had 4 mL of HBS buffer added and then was centrifuged for 10 minutes at 4,000 x g to a final volume of approximately 200 μL, this process was repeated 7 times, facilitating Cys removal and protein concentration. All aliquots were then combined and albumin concentration was determined with a BCA protein assay, following the manufacturer's protocol. In order to verify that no structural disulfide bonds were reduced and only the free Cys residue (Cys34) was reduced in the reduced sample, 5 μL of AlbSH was incubated with 5 μL of 50 mM maleimide in ammonium acetate buffer (pH 5). This was incubated at 50 °C for 15 minutes and then 1 μL of sample was mixed with 500 μL of 0.1% TFA and analyzed by LC-MS (**supplemental Fig. S3**). Pure S-Cys-Alb was obtained by following the same protocol but instead incubating 0.5 mM commercially prepared albumin with 1 mM Cys-Cys. All samples were stored at -80 °C until further analysis.

To eliminate interference from **Rxn. 2** during the rate law determinations, trace quantities of copper and other transition metals were minimized by pre-treating all buffers with Chelex 100 resin per the manufacturer's batch-wise instructions. Desferrioxamine (0.2 mM) was also added to the buffers employed in rate law determinations. To determine initial rates, pure AlbSH at concentrations ranging from 30-60 μM or pure S-Cys-Alb (30-90 μM) were incubated with free Cys-Cys or Cys (300-900 μM) at 23 °C, respectively (**supplemental Tables S1-S2**). A control

sample with no added Cys-Cys or Cys was also prepared to ensure that no artefactual oxidation or reduction occurred. Time courses for albumin oxidation and reduction were acquired by diluting 0.5 μL of a sample into 0.1% TFA to a final concentration of 1 μM albumin at various time points, which was then analyzed by LC-MS to provide time point concentrations of S-Cys-Alb and AlbSH. Time points were taken more frequently during the initial linear portion which yielded a slope that was used to determine the initial rate of reaction for each of the varying concentrations. The method of initial rates was then used to determine the reaction orders and rate constants for the disulfide-exchange oxidation and reduction of albumin (2). Slopes of log v_o vs. log reactant concentration (at several concentrations of the second reactant) were used to determine reaction orders and non-linear regression of the entire forward-reaction dataset and reverse reaction dataset was employed to determine the forward and reverse rate constants, respectively.

Rate Law-Based Model Verification in Actual Serum and Plasma

Immediately following collection, matched serum and K_2EDTA plasma from a healthy donor were each split into two 95-μL portions: One portion was spiked with Cys and Cys-Cys (in 5 μL of HBS buffer, pH 7.4) to increase the concentration of Cys by 12 μM and the concentration of Cys-Cys by 62 μM; the second portion was diluted by the same amount but without added Cys or Cys-Cys. These specimens were then incubated at 23 °C for 4 days, with numerous measurements of S-Cys-Alb collected initially and then at least once a day after Day 1. The data were then fit with the predictive model (using Wolfram Mathematica 10.2 or MatLab 2016), using the empirically determined initial concentrations of all species, determined as follows:

Measurement of Initial Reactant/Product Concentrations: Fresh aliquots of matched P/S samples were sent to ARUP Laboratories to determine concentrations of albumin (total), free copper and total copper. Initial fractions of S-Cys-Alb and AlbSH were determined using the S-Cys-Alb assay described above. These fractions were then converted to actual concentrations by multiplying by the total albumin concentration.

Cys-Cys concentration was determined from quadruplicate aliquots of serum and, separately, plasma using solid phase extraction (SPE) and gas chromatography-mass spectrometry (GC-MS) with procedures adapted from previously published reports (3-5). Calibration curve samples were prepared in 40 mg/ml albumin in HBS buffer (pH 7.4) with the addition of 0, 15, 30, 45, 60, or 90 μM of Cys-Cys. Three microliters of 2 mM Cys-Cys-d_4 internal standard in 1 M ammonium hydroxide and 1 M ammonium bicarbonate (pH 9.4) was combined with 96.5 μL of P/S and calibration curve samples, followed by 0.5 μL of 1 mM Cys-d_3 internal standard in 1 M ammonium hydroxide and 1 M ammonium bicarbonate (pH 9.4). Proteins were then immediately precipitated by first adding 200 μL of acetonitrile and then 300 μL of 0.2 M HCl which lowered the sample pH, minimizing disulfide exchange reactions. Samples were incubated on ice for 30 minutes and then centrifuged for 2 minutes at 13,000xg to remove protein precipitate. Sample supernatant was then stored on ice until ready for cation exchange SPE. The SPE cartridges were conditioned with 2 mL acetonitrile and then equilibrated with 2 mL of 0.1 M HCl. The entire sample was then loaded onto the cartridge and washed with 2 mL methanol. Samples were then eluted with 2 mL of 5% ammonium hydroxide in methanol into silanized glass test tubes and then dried down using a Savant SC250EXP SpeedVac Concentrator. After samples were dry, 50 μL of acetonitrile and 50 μL of N-methyl-N-(trimethylsilyl)trifluoroacetamide

(MSTFA) were added and then incubated for 30 minutes at 85 °C. Samples were then loaded into GC-MS autosampler vials and injected onto the GC-MS.

GC-MS analysis was carried out on an Agilent Model A7890 gas chromatograph (equipped with a CTC PAL autosampler) coupled to a Waters GCT (time-of-flight) mass spectrometer. One microliter of the sample was injected in split mode onto an Agilent split-mode liner that contained a small plug of silanized glass wool. The injector temperature was set at 280 °C and the split ratio was 5:1. The carrier gas was regulated in constant flow mode at 0.8 mL/min. The capillary column was a 30-m fused silica DB-5MS with a 0.25 μM film thickness and 0.250 mm inner diameter. The temperature program was started at 100 °C with initial holding for 1 minute and was increased at the rate of 10 °C/min to 300 °C and held for 3 minutes. The temperature was then increased 30 °C/min to 325 °C, with final holding of 3 minutes. The temperature of the transfer line to the MS was 280 °C. Mass spectra were obtained by standard (70 eV) electron ionization scanning from *m/z* 40 to 800 at a spectral accumulation rate of 0.09 seconds/spectrum.

Quantification was done by integrating summed extracted ion chromatogram (XIC) peak areas, using QuanLynx software. The peaks were integrated automatically and verified manually. The extracted ions (± 0.15 *m/z* units) used for quantification were: Cys-Cys (*m/z* 411.1 and 232.1), Cys-Cys-d_4 (*m/z* 415.1 and 234.1), Cys (*m/z* 220.1, 232.1, and 322.1), Cys-d_2 (which results from reduction of Cys-Cys-d_4 during derivatization as described below; *m/z* 222.1, 234.1, and 324.1) and Cys-d_3 (*m/z* 223.1, 235.1, and 325.1). All summed XIC peak integrals were exported to a spreadsheet for further analysis.

Cys-Cys is partially reduced to Cys during the derivatization step that is necessary to facilitate analysis by GC-MS. As such, Cys ions were used in the quantification of Cys-Cys. However, in order to use Cys in this quantification scheme it was necessary to subtract out any signal that was due to the endogenous Cys. This was done by spiking in 5 μM Cys-d_3 into each sample, which is equivalent to the concentration of endogenous Cys in P/S samples (as explained in the next paragraph and illustrated in **supplemental Fig. S4**). The magnitude of the summed XIC areas from Cys-d_3 was then assumed to represent the magnitude of the summed XIC areas from endogenous Cys. Thus, the total area of the summed XICs from Cys-d_3 was subtracted from the summed XIC areas of endogenous Cys. All residual summed XIC peak area from Cys ions was then considered to be derived from Cys-Cys. Prior to making this calculation, however, isotopic overlap between natural forms of Cys ions and their d_2-labeled internal standard counterparts was corrected for based on the known isotopic distribution of each fragment ion employed for quantification. This isotope correction procedure was analogous to that which has previously been employed and widely used elsewhere (6, 7).

Given the initial concentrations of AlbSH, S-Cys-Alb, free and total copper, and Cys-Cys determined above, all rate law models with initial Cys concentrations anywhere within the physiologically observed range (8, 9) revealed that by the time plasma and serum were separated from whole blood, the concentration of Cys in P/S had equilibrated to a steady state concentration of ~ 5 μM (**supplemental Fig. S4**). Given this information and the fact that, relative to Cys-Cys, Cys contributes ≤ 5% of the total Cys equivalents in P/S, an initial Cys concentration of 5 μM was assumed in all kinetic models.

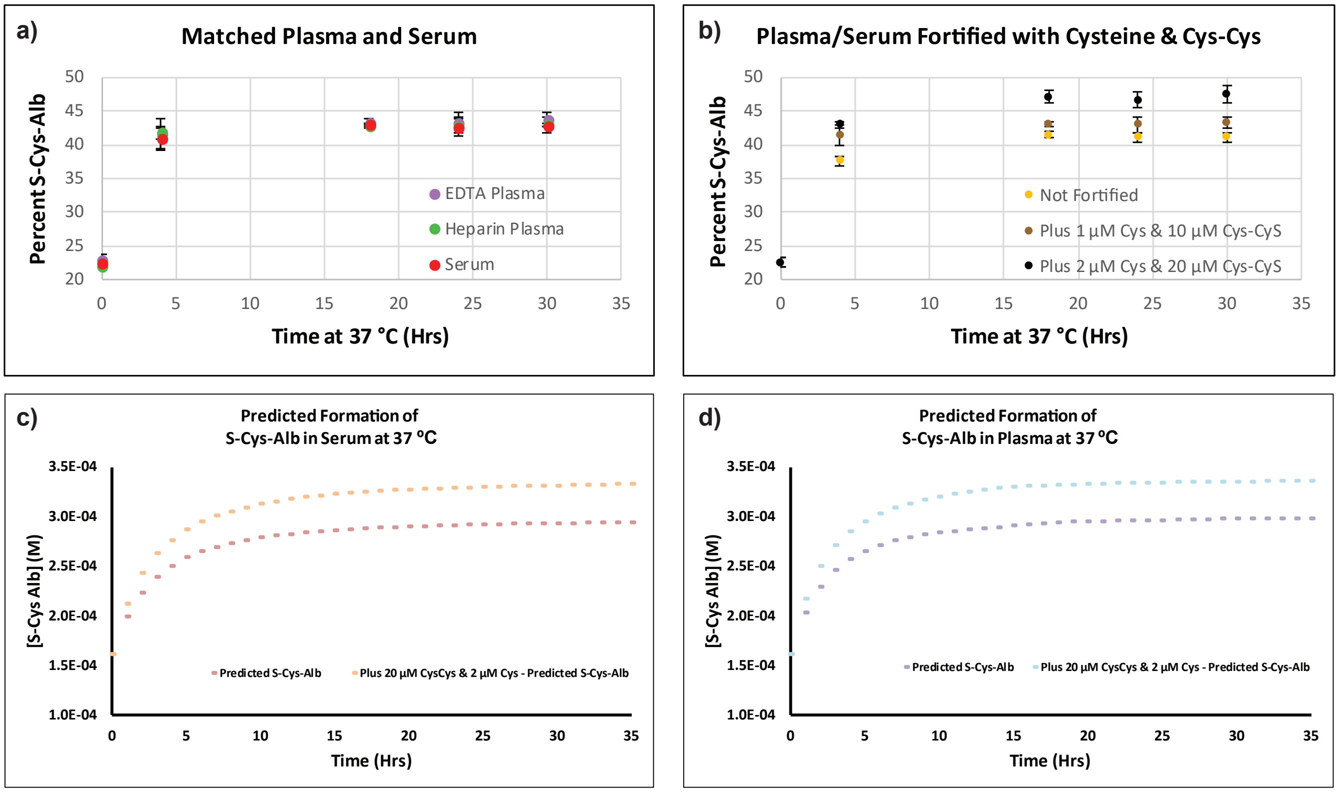

Figure S1: Determination of the time required at 37 °C to maximize S-Cys-Alb in P/S samples. Empirical evaluation (panels a-b): a) Nine-microliter aliquots of matched K_2EDTA plasma, sodium heparin plasma, and serum from a healthy donor were incubated in closed 0.6-mL test tube at 37 °C for up to 30 hrs. No significant differences were found between plasma and serum or between the two types of plasma. No changes were observed after 18 hrs at 37 °C. n = 3 per data point; error bars represent SD. b) Each P/S sample was divided into 3 portions; one was left unmodified, to the second was added 1 µM Cys and 10 µM Cys-Cys, and to the third was added 2 µM Cys and 20 µM Cys-Cys. The addition of Cys and Cys-Cys results in an increase in the maximum value of S-Cys-Alb observed, but does not increase the time frame required to reach the maximum obtainable value of S-Cys-Alb at 37 °C. Data from both types of plasma and serum were pooled and are displayed together as mean ± SD (n = 9 per data point).
Panels c-d provide theoretical trajectories for S-Cys-Alb formation in P/S based on the rate law model described for Fig. 6. These also reach a plateau by 18 hrs. Input parameters include age-weighted population averages for AlbSH (484 µM) and S-Cys-Alb (484 µM) (10,11), Cys-Cys (65 µM) (8,9), Cys (5 µM—see Fig. S4); the population average for total Cu(II) (18.7 µM) (12)—all of which is assumed catalytically available in plasma and 50% of which is assumed to be catalytically available in serum (see main text for rationale); k_3 (0.32 M^{-1} s^{-1}), K_y (5.1 x 10^{-6} M) and K_z (8.8 x 10^{-4} M) as determined by Kachur et al. at 37 °C (13); and k_1 (0.6 M^{-1} s^{-1}) and k_2 (6.6 M^{-1} s^{-1}) as recently determined by Bocedi et al. at 37 °C (14).

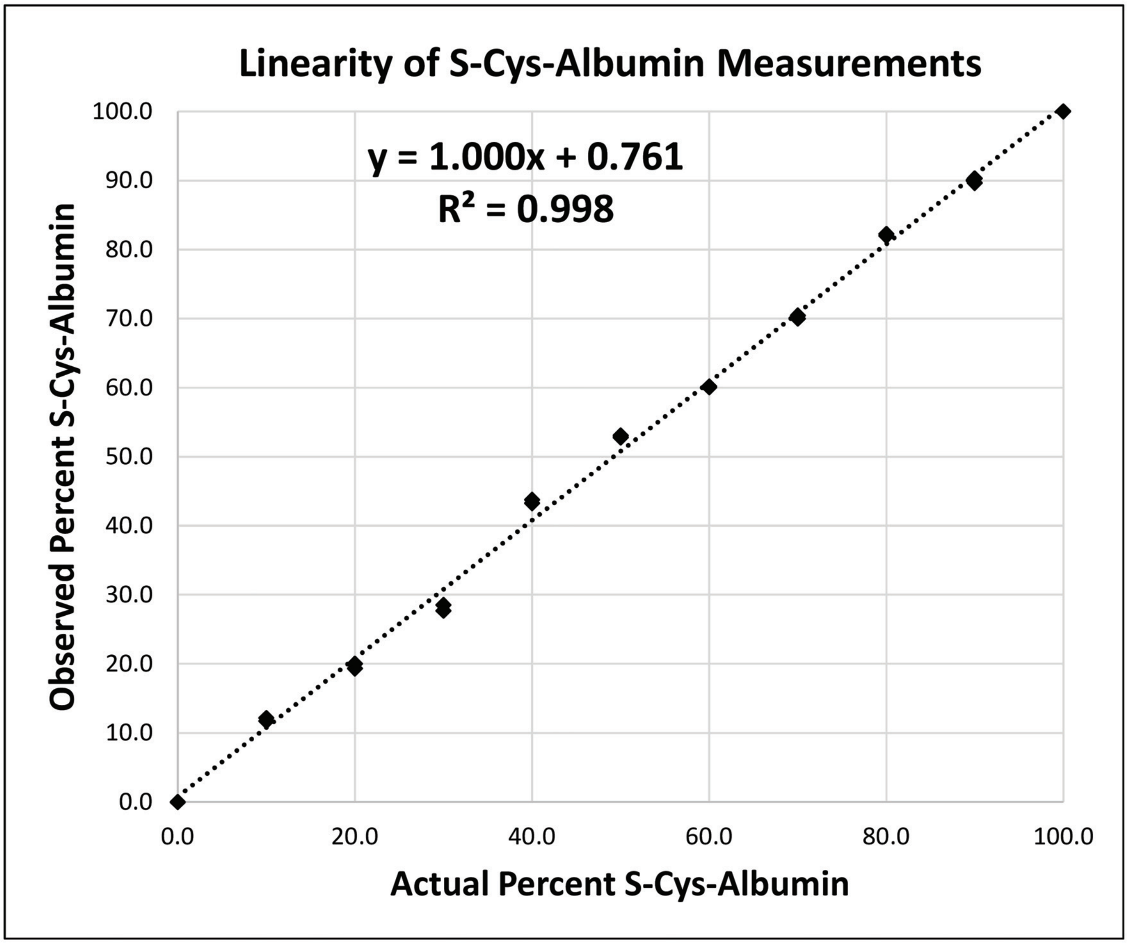

Figure S2: Linearity of S-Cys-Albumin measurements.

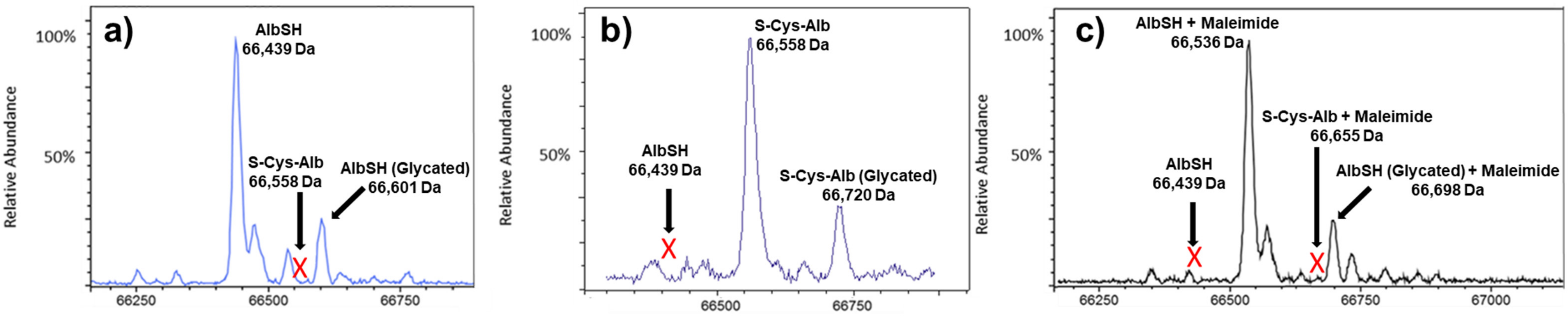

Figure S3: Charge deconvoluted ESI-mass spectra of the a) fully reduced and b) fully oxidized (S-cysteinylated) albumin employed for determination of the forward and reverse rate laws of the reactions shown in Rxn 1. c) To verify that no structural disulfide bonds were reduced in the former case, the sample was alkylated with maleimide. The mass shift of exactly +97 Da, with no peaks at +2*97 Da (66,633 Da, corresponding to two alkylation events) or +3*97 Da etc. indicate that the reduced albumin possessed only a single free Cys residue as expected.

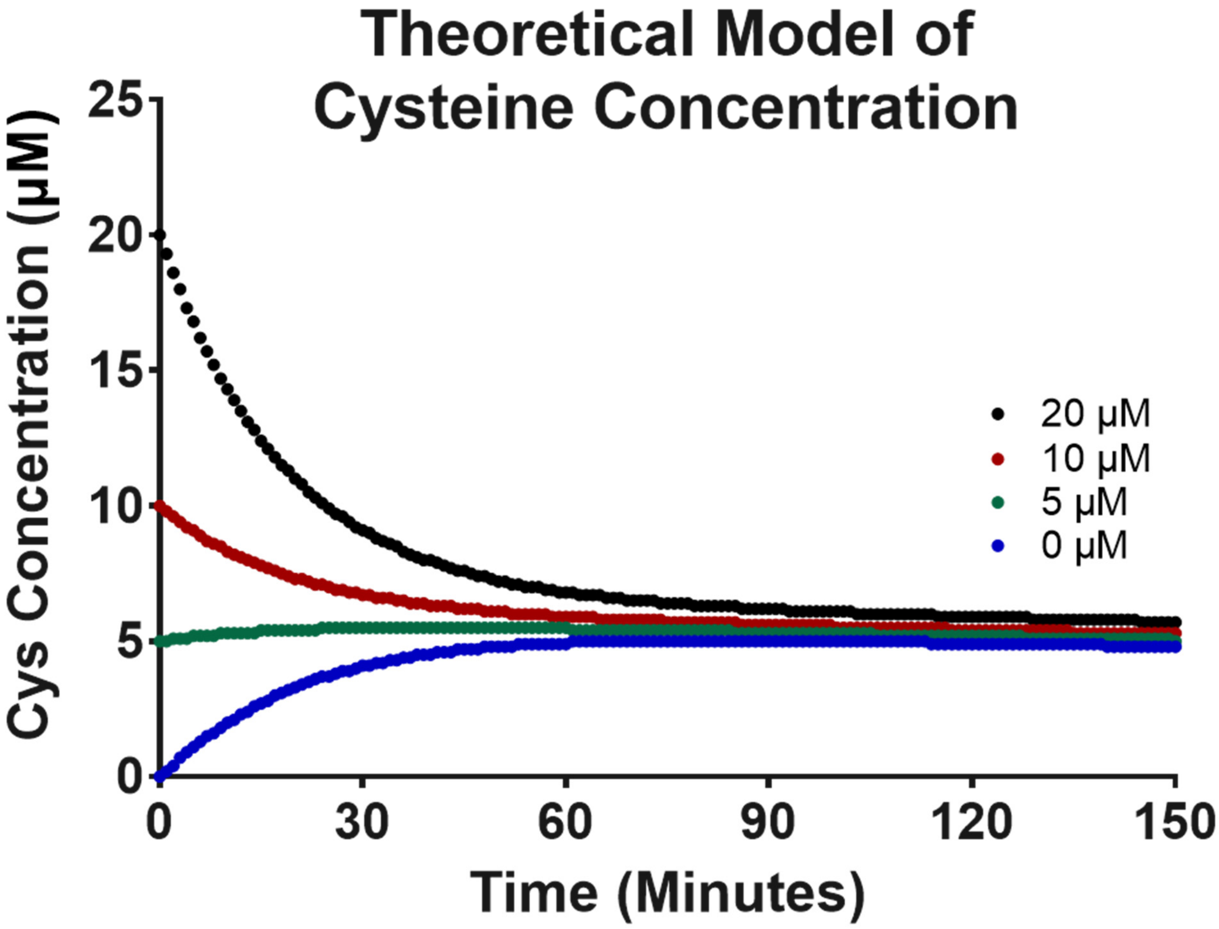

Figure S4: Reaction kinetics models based on the empirically determined rate law (Eqns. 5-8) illustrating that by the time serum or plasma are processed from whole blood (~ 60 min), the concentration of Cys approaches a near steady-state concentration of about 5 µM—regardless of whether or not the initial concentration started at the low or high end of physiological Cys concentrations observed in human plasma (8, 9).

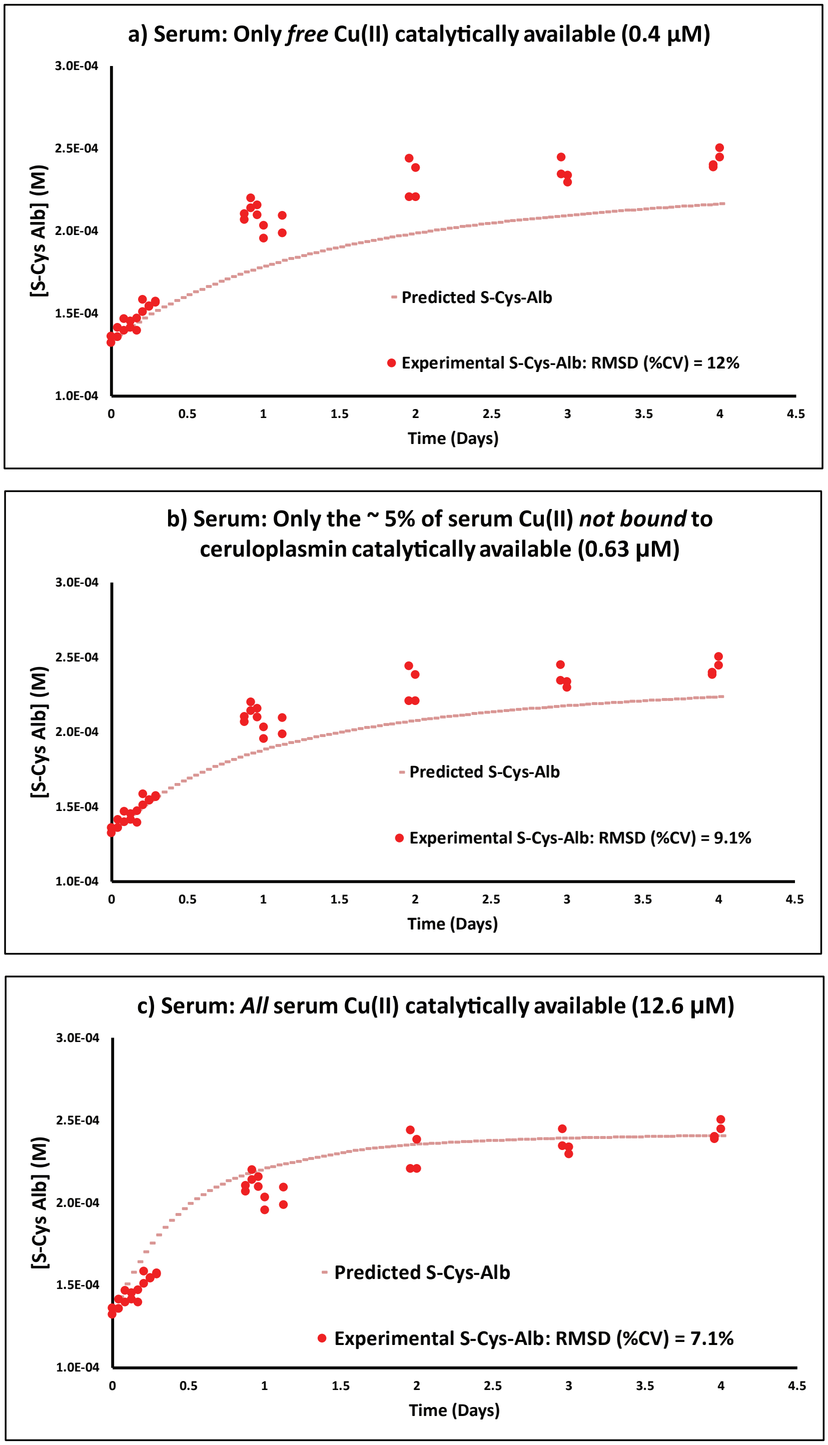

Figure S5. Kinetics simulations in which a) only free serum Cu(II) (0.4 µM), b) the ~5% of serum Cu(II) not bound to ceruloplasmin (0.63 µM), and c) all Cu(II) in serum (12.6 µM) is assumed to be catalytically available.

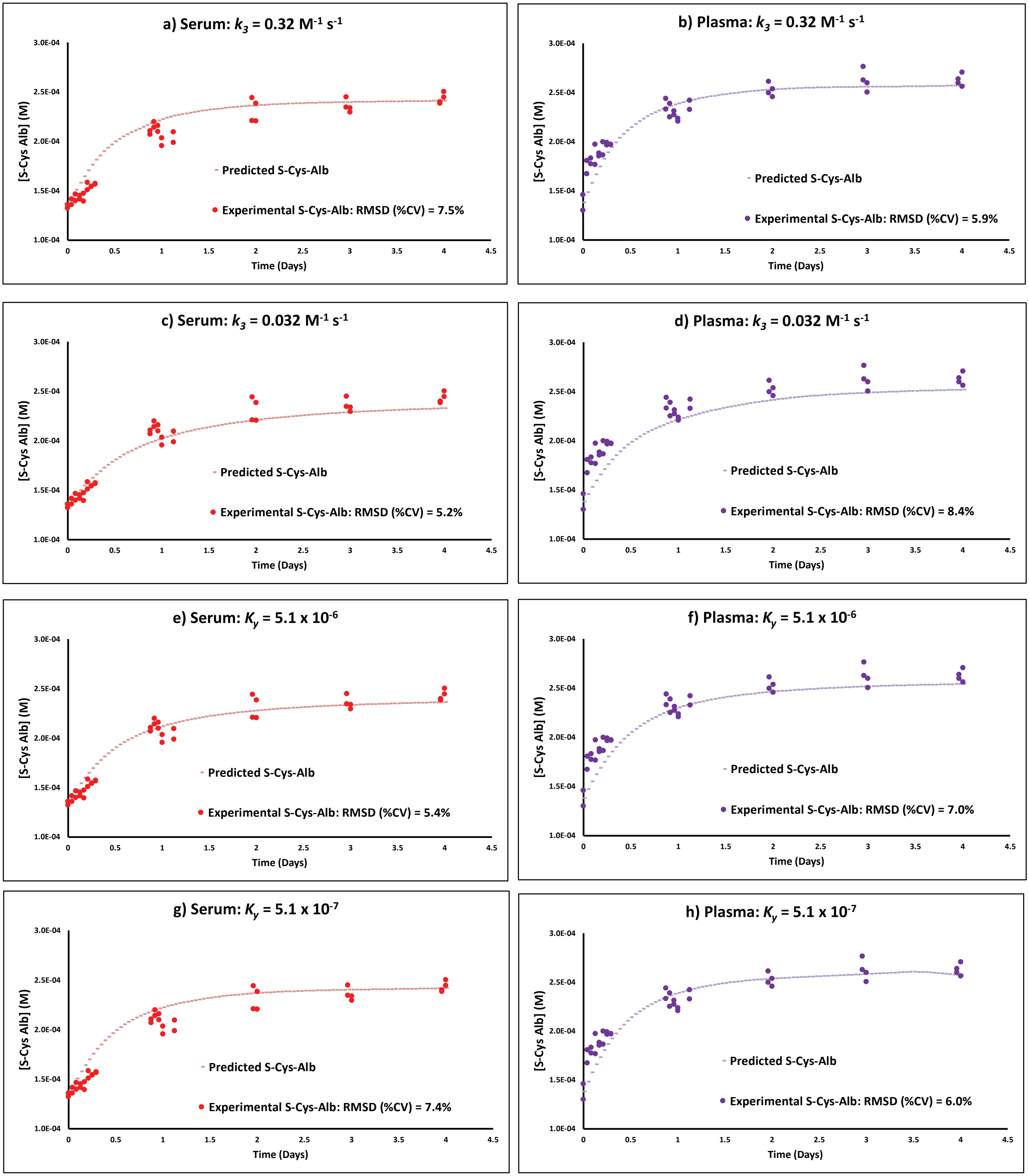

Figure S6 (continued on next page)

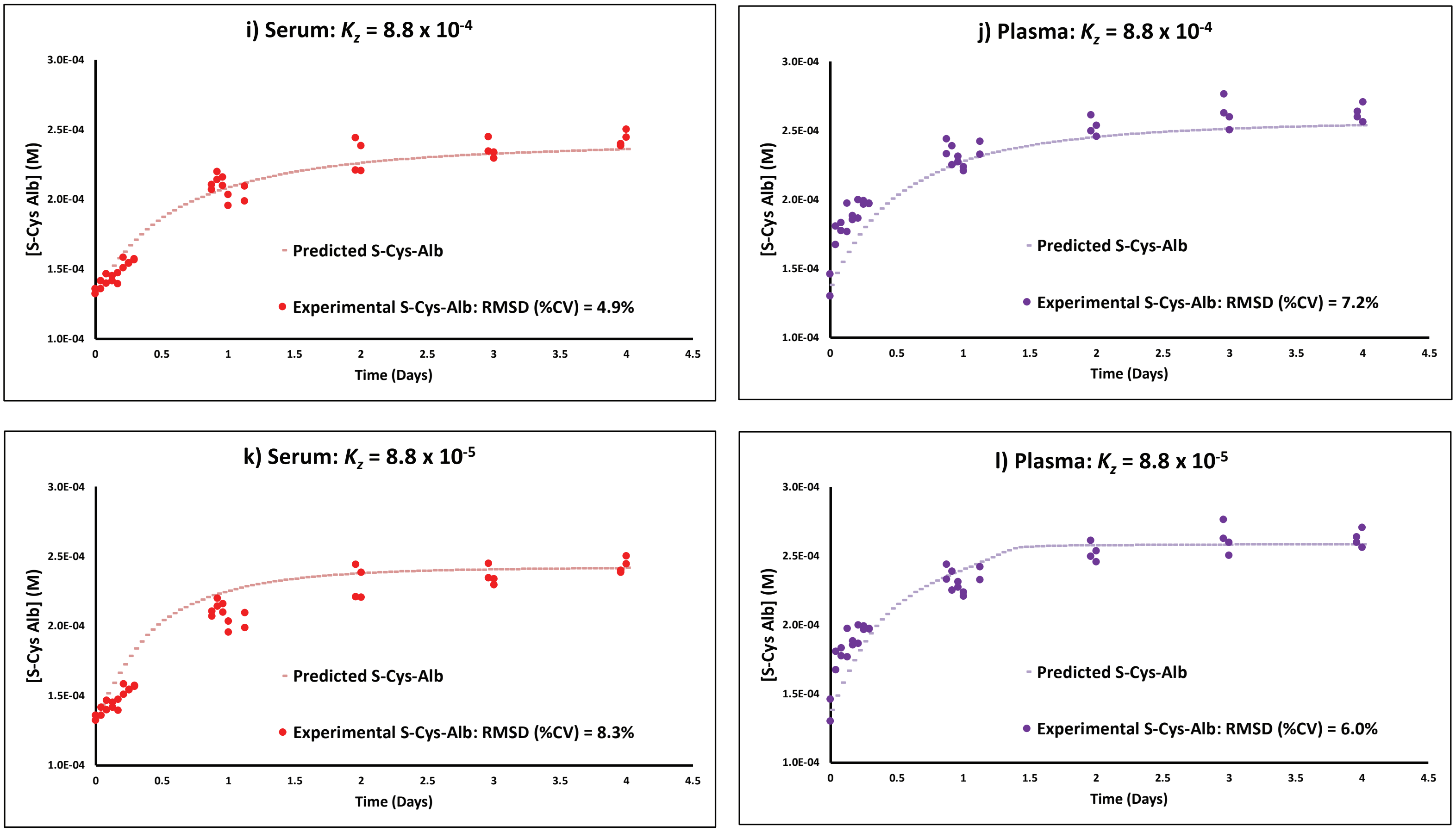

Figure S6. Kinetics simulations in which the Rxn 2 rate law constants k_3, K_y and K_z are individually changed to their 37 °C values or 10x below these values. a-b) Serum and plasma, respectively, where k_3 = 0.32 M^{-1} s^{-1}, the value established by Kachur et al. (13) at 37 °C; c-d) k_3 = 0.032 M^{-1} s^{-1}; e-f) K_y = 5.1 x 10^{-6} M, the value established by Kachur et al. (13) at 37 °C; g-h) K_y = 5.1 x 10^{-7} M; i-j) K_z = 8.8 x 10^{-4} M, the value established by Kachur et al. (13) at 37 °C; k-l) K_z= 8.8 x 10^{-5} M.

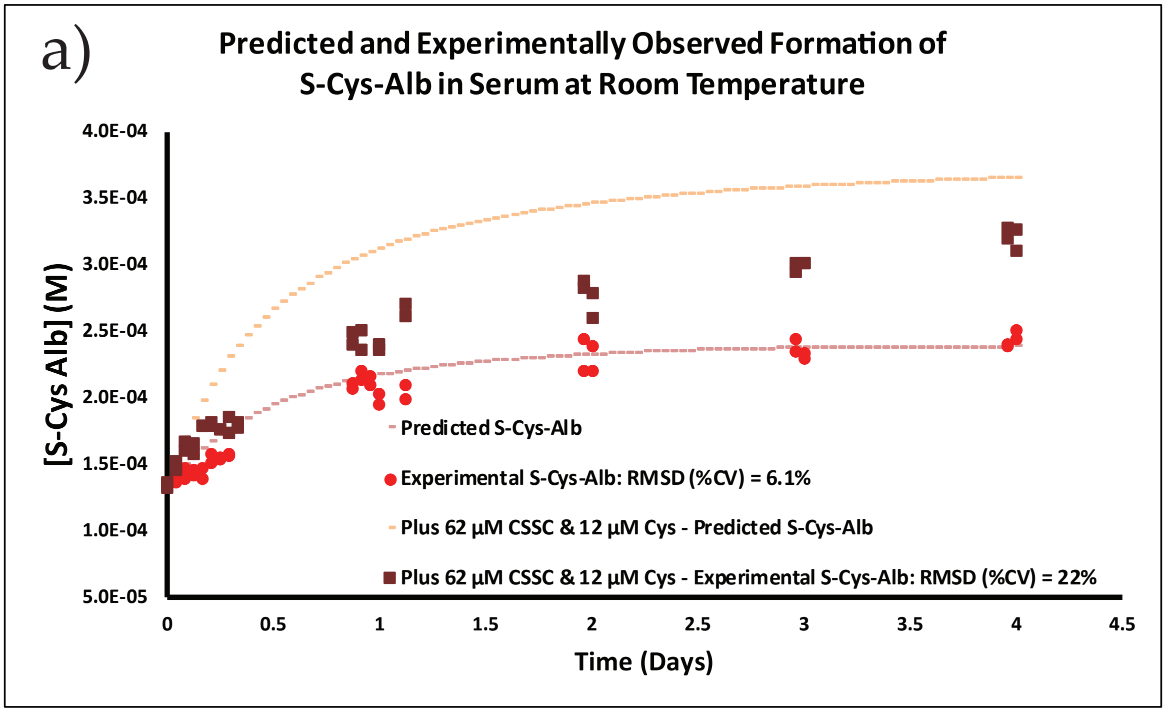

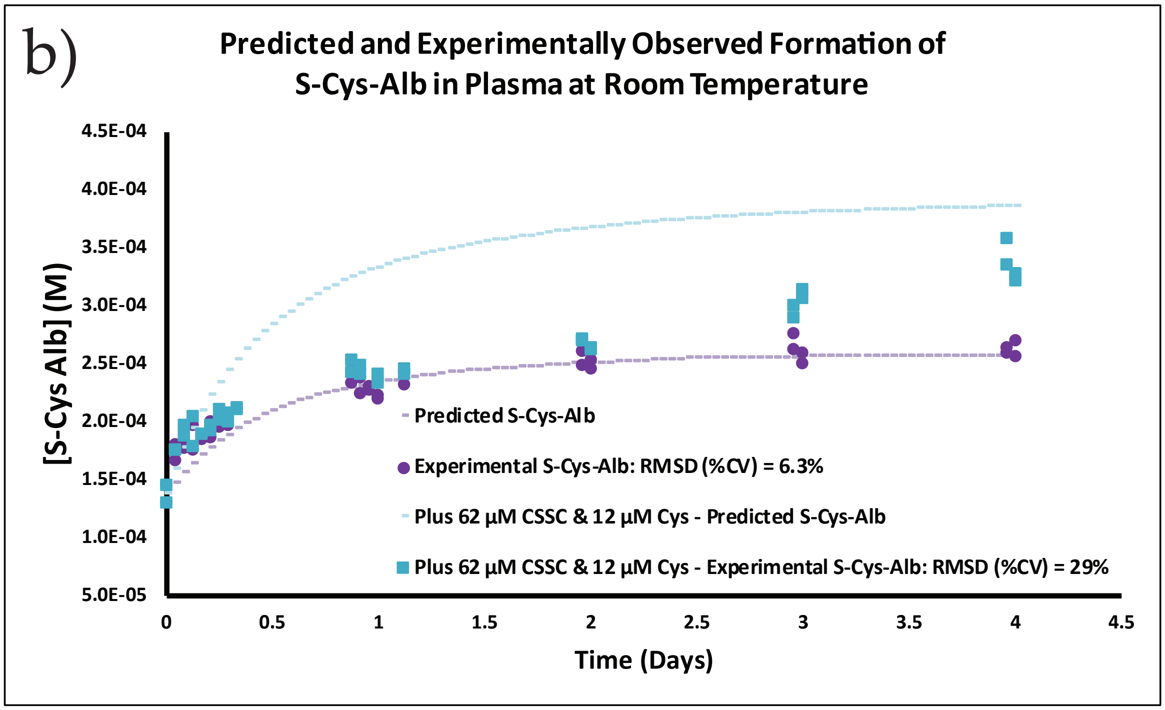

Figure S7: Observed and rate law model-predicted formation of S-Cys-Alb in matched a) serum and b) K_2EDTA plasma from a healthy donor. Circles represent natural, unfortified serum or plasma containing initially measured concentrations of AlbSH = 609 µM (serum) or 605 µM (plasma); S-Cys-Alb = 134 µM (serum) or 138 µM (plasma); Cys-Cys = 52 µM (serum) or 58 µM (plasma); Cys = 5 µM (inferred, not measured, see Results text and supplemental Fig. S4); and Cu(II) = 12.6 µM. Squares represent aliquots of the same samples into which extra Cys-Cys and Cys were fortified, bringing the final concentration of Cys-Cys to 114 µM (serum) or 120 µM (plasma) and Cys to 17 µM (serum & plasma). Dashed lines represent rate model-predicted trajectories for S-Cys-Alb formation based on numerical solutions to Eqns. 5-8 employing the rate and equilibrium constant parameters described in the main text. The poor model fit for samples fortified with extra Cys-Cys and Cys appears to be due to the concentration of dissolved oxygen, $[O_{2(aq)}]$, becoming rate limiting under these fortified conditions (supplemental Fig. S8). Cys-Cys is abbreviated as CSSC in the legends.

a) ***Chemical Reactions***

$$AlbSH + Cys\text{-}Cys \underset{k_2}{\overset{k_1}{\rightleftharpoons}} SCysAlb + Cys \quad (1)$$

$$2Cys + O_2 \xrightarrow[k_3]{Cu(II)} Cys\text{-}Cys + H_2O_2 \quad (2)$$

$$\left\{\begin{array}{ll} Cys + Cu(II) \xrightarrow{k_x} 1/2\, Cys\text{-}Cys + Cu(I) & (10) \\ Cu(I) + O_2 \xrightarrow{k_4} Cu(II) + O_2^{\cdot-} & (11) \end{array}\right\}$$

Rate Equations

$$\frac{d[AlbSH]}{dt} = -k_1[AlbSH][Cys\text{-}Cys] + k_2[SCysAlb][Cys] \quad (5)$$

$$\frac{d[Cys\text{-}Cys]}{dt} = -k_1[AlbSH][Cys\text{-}Cys] + k_2[SCysAlb][Cys] + \frac{k_3[Cu(II)][Cys]}{K_z\left(1 + \frac{K_y}{[Cys]}\right) + [Cys]} \quad (6)$$

$$\frac{d[SCysAlb]}{dt} = k_1[AlbSH][Cys\text{-}Cys] - k_2[SCysAlb][Cys] \quad (7)$$

$$\frac{d[Cys]}{dt} = k_1[AlbSH][Cys\text{-}Cys] - k_2[SCysAlb][Cys] - 2\left(\frac{k_3[Cu(II)][Cys]}{K_z\left(1 + \frac{K_y}{[Cys]}\right) + [Cys]}\right) \quad (8)$$

$$\frac{d[Cu(II)]}{dt} = -2\left(\frac{k_3[Cu(II)][Cys]}{K_z\left(1 + \frac{K_y}{[Cys]}\right) + [Cys]}\right) + k_4[Cu(I)][O_2] \quad (12)$$

$$\frac{d[Cu(I)]}{dt} = 2\left(\frac{k_3[Cu(II)][Cys]}{K_z\left(1 + \frac{K_y}{[Cys]}\right) + [Cys]}\right) - k_4[Cu(I)][O_2] \quad (13)$$

$$\frac{d[O_2]}{dt} = k_{O_2} - k_4[Cu(I)][O_2] \quad (14)$$

Figure S8. Kinetics model and simulations that take $O_{2(aq)}$ into account.

Part a) Chemical and mathematical rate equations. Rxns. 10-11 break apart Rxn. 2 to show how $O_{2(aq)}$ recycles Cu(I) into Cu(II) and allows Cu(II) to serve as the reaction catalyst. The rate law described by Kachur et al. (13) for Rxn. 2 omits O_2 (i.e., assumes it is not rate limiting) and takes into account the equilibrium binding affinities for the first and second Cys liganding to Cu(II). Thus, strictly speaking, it is actually the rate law for Rxn. 10—but one in which the Cu(I) produced is assumed to be immediately recycled back to Cu(II). When $O_{2(aq)}$ is in short supply, Rxn. 11 can become rate limiting and must be taken into account in the model.

Taking $O_{2(aq)}$ into account requires three additional differential equations (Eqns. 12-14), two additional reaction components ($O_{2(aq)}$ and Cu(I)) and two additional rate constants (k_4 and $\boldsymbol{k_{O_2}}$). Simulations based on this model (see *Fig. S8 Part b*) should be considered speculative because not all of these parameters are known: $O_{2(aq)}$ was estimated at 30% saturation (70 µM) and Cu(I) was assumed to initially exist only in trace quantities (i.e., < 1% of total Cu or 0.1 µM). k_4 will vary depending on how Cu(II) is bound in P/S. A value of 200 $M^{-1}\ s^{-1}$ was employed based on the known rate constant for the reaction of $O_{2(aq)}$ with the Fe(II)-EDTA complex (15) (that for the Cu(I)-EDTA complex is unknown). $\boldsymbol{k_{O_2}}$ is a constant that describes the rate at which $O_{2(g)}$ from the headspace above the P/S sample dissolves, becoming how $O_{2(aq)}$. It was estimated at 1 x 10^{-10} M/s.

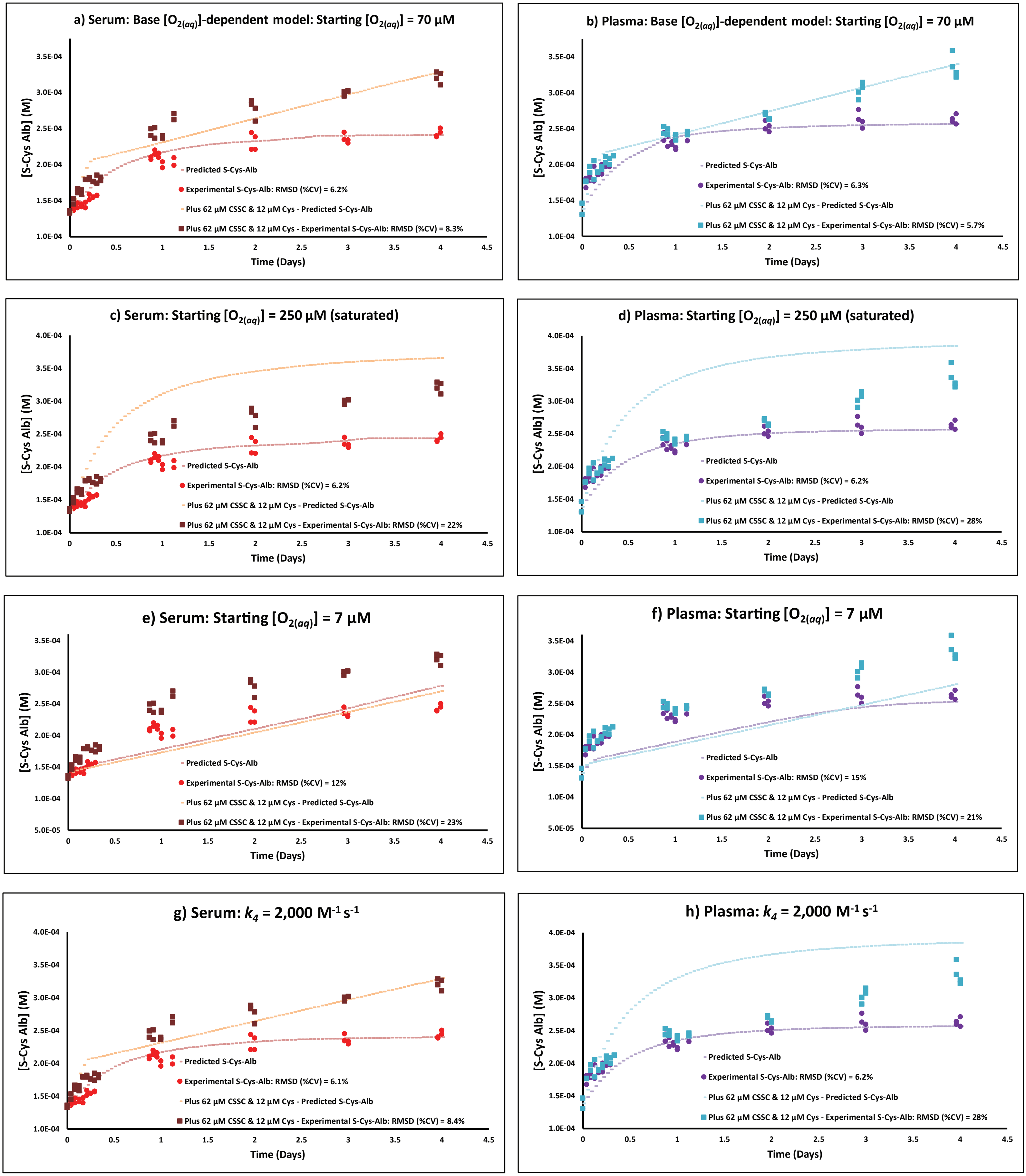

Figure S8 (Part b, continued on next page)

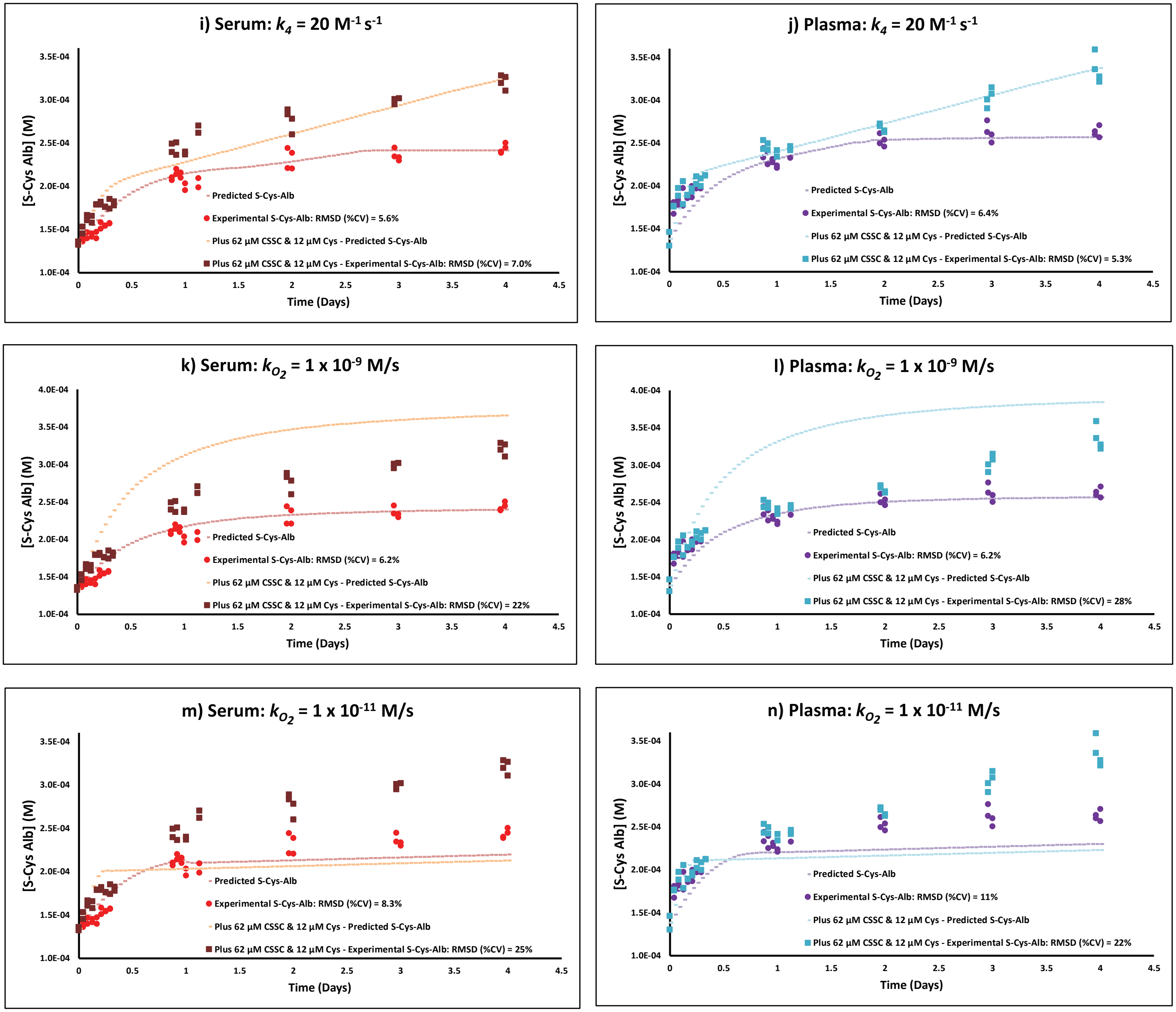

Figure S8. Kinetics model and simulations that take $O_{2(aq)}$ into account.
Part b) Model predictions (lines), overlaid on actual results (symbols), that take into account best estimates for the additional parameters (reaction components and rate constants) required for an $O_{2(aq)}$–dependent model as described in *Part a*. Here, panels a-b, correspond to serum and K_2EDTA plasma with starting $O_{2(aq)}$ = 70 µM, respectively. All other parameters in the model exist as described in Fig. 6. Additional panels vary the starting concentration of one reaction component or one rate constant at a time (generally -10x or +10x unless physically unreasonable) to evaluate its effect on the model. c-d) Starting $O_{2(aq)}$ = 250 µM (saturation); e-f) Starting $O_{2(aq)}$ = 7 µM; g-h) k_4 = 2,000 M^{-1} s^{-1} ; i-j) k_4 = 20 M^{-1} s^{-1} ; k-l) k_{O_2} = 1 x 10^{-9} M/s; m-n) k_{O_2} = 1 x 10^{-11} M/s (This simulates placing P/S into a nitrogen (low $O_{2(g)}$) atmosphere following initial processing (which permits a modest initial concentration of $O_{2(aq)}$ to develop in the sample—i.e., ~ 70 µM). The trajectory difference between the predicted and observed formation of S-Cys-Alb matches, approximately, the difference between the unfortified plasma sample incubated under nitrogen and the one incubated under air in Fig. 7.) Cys-Cys is abbreviated as CSSC in the legends.

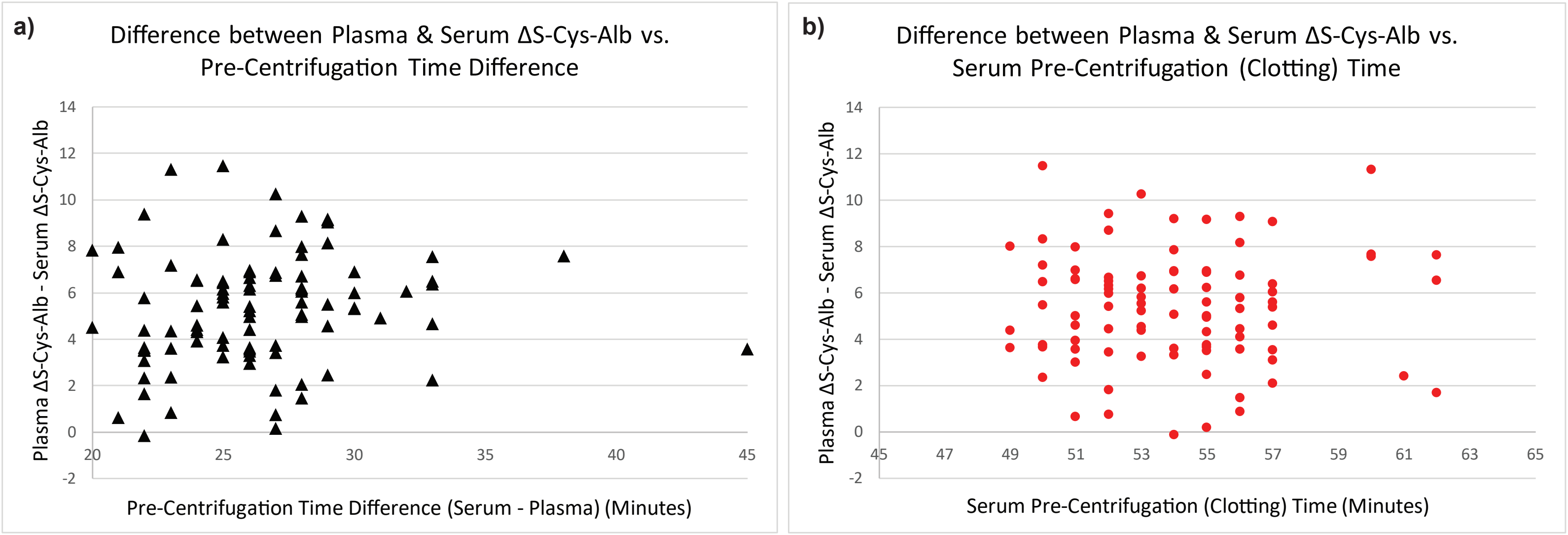

Figure S9. The *difference* between *matched* plasma and serum sample ΔS-Cys-Alb values was not correlated to a) the pre-centrifugation time difference between plasma and serum or b) serum pre-centrifugation (clotting) time. ($p > 0.05$ in both cases; Spearman correlation).

Table S1. Initial concentrations employed for determining the rate law for S-cysteinylation (oxidation) of albumin (AlbSH) by cystine (Cys-Cys). Values listed inside the pivot table are initial rates in units of M/s.

	$[Cys\text{-}Cys]_0$ (M)				
$[AlbSH]_0$ (M)	0.0003	0.00045	0.0006	0.00075	0.0009
0.00003	6.68E-10	9.35E-10	1.31E-09	1.33E-09	2.38E-09
0.000045	1.61E-09	1.85E-09	2.67E-09	3.52E-09	4.67E-09
0.00006	1.69E-09	2.21E-09	2.8E-09	4.06E-09	4.88E-09
0.000075	2.25E-09	2.84E-09	4.77E-09	5.92E-09	6.87E-09
0.00009	2.88E-09	5.33E-09	5.12E-09	7.9E-09	8.61E-09

Table S2. Initial concentrations employed for determining the rate law for reduction of S-cysteinylated albumin (S-Cys-Alb) by cysteine (Cys). Values listed inside the pivot table are initial rates in units of M/s.

	$[Cys]_0$ (M)				
$[S\text{-}Cys\text{-}Alb]_0$ (M)	0.0003	0.00045	0.0006	0.00075	0.0009
0.00003	3.008E-08	4.84E-08	6.0667E-08	7.79E-08	9.75E-08
0.000045	4.679E-08	6.091E-08	7.608E-08	1.12E-07	1.33E-07
0.00006	8.413E-08	9.616E-08	1.1294E-07	1.26E-07	1.67E-07

Table S3: Starting reactant and product concentrations for Fig. 10 and Table 1. [a,b]

	Alb_{tot}		AlbSH		S-Cys-Alb		Cys-Cys		Cys		Cu		Fraction S-Cys-Alb		ΔS-Cys-Alb (as fraction)	
	Plasma	Serum	Plasma	Serum	Plasma	Serum	Plasma	Serum	Plasma	Serum	Plasma	Serum	Plasma	Serum	Plasma	Serum
**Average P/S ΔS-Cys-Albumin**	646	646	462	469	184	177	65.0	47.5	5	5	18.7	9.35	0.285	0.274	0.209	0.155
**High P/S ΔS-Cys-Albumin (Avg. Rate)** [c]	540	540	386	392	154	148	73.9	56.5	5	5	18.7	9.35	0.285	0.274	0.283	0.218
**Low P/S ΔS-Cys-Albumin (Avg. Rate)**	752	752	538	546	214	206	48.3	31.7	5	5	18.7	9.35	0.285	0.274	0.135	0.091
**High P/S ΔS-Cys-Albumin (Slow Rate)**	540	540	333	336	207	204	73.9	56.5	5	5	9.5	4.75	0.384	0.378	0.283	0.218
**High P/S ΔS-Cys-Albumin (Fast Rate)**	540	540	440	448	100	92	73.9	56.5	5	5	27.9	13.95	0.185	0.170	0.283	0.218
**Low P/S ΔS-Cys-Albumin (Fast Rate)**	752	752	613	624	139	128	48.3	31.7	5	5	27.9	13.95	0.185	0.170	0.135	0.091
**Low P/S ΔS-Cys-Albumin (Slow Rate)**	752	752	463	468	289	284	48.3	31.7	5	5	9.5	4.75	0.384	0.378	0.135	0.091

[a] All concentrations are in units of micromolar

[b] Fraction S-Cys-Alb and ΔS-Cys-Alb are based on the population values determined here. Total albumin (Alb_{tot}) is based on the U.S. age distribution weighted population average for serum albumin (10,11). Cu(II) is based on the U.S. population average concentration of copper in serum (12); values for the kinetics model are halved in serum due to the sequestration of copper in ceruloplasmin as explained in the main text. Cys is fixed at 5 micromolar as explained in the main text. Cys-Cys was set based on Alb_{tot} and the targeted ΔS-Cys-Alb (as fraction) value. AlbSH and S-Cys-Alb were set based on the targeted Fraction S-Cys-Alb value.

[c] High/Low ΔS-Cys-Albumin values are based on 2 SDs above/below the population mean. Fast/slow rates are based on copper concentrations that are 2 SDs above/below the population mean *and* Fraction S-Cys-Alb that is 2 SDs below/above the population mean.

References Cited in Supplemental Data

1. Borges, C. R., Rehder, D. S., Jensen, S., Schaab, M. R., Sherma, N. D., Yassine, H., Nikolova, B., and Breburda, C. (2014) Elevated Plasma Albumin and Apolipoprotein A-I Oxidation under Suboptimal Specimen Storage Conditions. *Molecular & cellular proteomics : MCP* 13, 1890-1899

2. Atkins, P. W. (1994) *Physical Chemistry*, 5th Ed., W. H. Freeman and Company, Chapter 25 - *The rates of chemical reactions*; New York, NY

3. Orsak, T., Smith, T. L., Eckert, D., Lindsley, J. E., Borges, C. R., and Rutter, J. (2012) Revealing the Allosterome: Systematic Identification of Metabolite-Protein Interactions. *Biochemistry-Us* 51, 225-232

4. Johnson, J. M., Strobel, F. H., Reed, M., Pohl, J., and Jones, D. P. (2008) A rapid LC-FTMS method for the analysis of cysteine, cystine and cysteine/cystine steady-state redox potential in human plasma. *Clin Chim Acta* 396, 43-48

5. Ohie, T., Fu, X., Iga, M., Kimura, M., and Yamaguchi, S. (2000) Gas chromatography-mass spectrometry with tert.-butyldimethylsilyl derivation: use of the simplified sample preparations and the automated data system to screen for organic acidemias. *J Chromatogr B Biomed Sci Appl* 746, 63-73

6. Liebisch, G., Lieser, B., Rathenberg, J., Drobnik, W., and Schmitz, G. (2004) High-throughput quantification of phosphatidylcholine and sphingomyelin by electrospray ionization tandem mass spectrometry coupled with isotope correction algorithm. *Biochim Biophys Acta* 1686, 108-117

7. Liebisch, G., Lieser, B., Rathenberg, J., Drobnik, W., and Schmitz, G. (2005) Erratum to "High-throughput quantification of phosphatidylcholine and sphingomyelin by electrospray ionization tandem mass spectrometry coupled with isotope corrections algorithm" [Biochimica et Biophysics Acta, 1686 (2004) 108–117]. *Biochim Biophys Acta* 1734, 86-89

8. Jones, D. P., Mody, V. C., Jr., Carlson, J. L., Lynn, M. J., and Sternberg, P., Jr. (2002) Redox analysis of human plasma allows separation of pro-oxidant events of aging from decline in antioxidant defenses. *Free Radic Biol Med* 33, 1290-1300

9. Blanco, R. A., Ziegler, T. R., Carlson, B. A., Cheng, P. Y., Park, Y., Cotsonis, G. A., Accardi, C. J., and Jones, D. P. (2007) Diurnal variation in glutathione and cysteine redox states in human plasma. *Am J Clin Nutr* 86, 1016-1023

10. United States Center for Disease Control and Prevention, National Health and Nutrition Examination Survey (NHANES), 2015-2016.

11. Weaving, G., Batstone, G. F., and Jones, R. G. (2016) Age and sex variation in serum albumin concentration: an observational study. *Annals of clinical biochemistry* 53, 106-111

12. United States Center for Disease Control and Prevention, National Health and Nutrition Examination Survey (NHANES), 2013-2014.

13. Kachur, A. V., Koch, C. J., and Biaglow, J. E. (1999) Mechanism of copper-catalyzed autoxidation of cysteine. *Free Radic Res* 31, 23-34

14. Bocedi, A., Cattani, G., Stella, L., Massoud, R., and Ricci, G. (2018) Thiol disulfide exchange reactions in human serum albumin: the apparent paradox of the redox transitions of Cys34. *FEBS J* 285, 3225-3237

15. Seibig, S., and vanEldik, R. (1997) Kinetics of [Fe-II(edta)] oxidation by molecular oxygen revisited. New evidence for a multistep mechanism. *Inorg Chem* 36, 4115-4120
